# CD38 expression by neonatal human naive CD4^+^ T cells shapes their distinct metabolic and tolerogenic properties

**DOI:** 10.1172/JCI200062

**Published:** 2026-02-26

**Authors:** Laura R. Dwyer, Andrea M. DeRogatis, Sean Clancy, Victoire Gouirand, Charles Chien, Elizabeth E. Rogers, Scott P. Oltman, Laura L. Jelliffe-Pawlowski, Theo van den Broek, Femke van Wijk, Susan V. Lynch, Rachel L. Rutishauser, Allon Wagner, Alexis J. Combes, Tiffany C. Scharschmidt

**Affiliations:** 1 Biomedical Sciences Graduate Program, and; 2 Department of Dermatology, UCSF, San Francisco, USA.; 3 Department of Electrical Engineering and Computer Sciences, and; 4 Center for Computational Biology, University of California, Berkeley, Berkeley, USA.; 5 Healthy Outcomes of Pregnancy for Everyone Research Consortium,; 6 Department of Pediatrics,; 7 Department of Epidemiology and Biostatistics,; 8 Department of Global Health Sciences, and; 9 California Preterm Birth Initiative, UCSF, San Francisco, USA.; 10 Rory Meyers College of Nursing, New York University, New York, New York, USA.; 11 EGG Healthy Pregnancy, San Francisco, California, USA.; 12 Center for Translational Immunology, University Medical Centre Utrecht, Utrecht University, Netherlands.; 13 Division of Gastroenterology,; 14 Benioff Center for Microbiome Medicine, and; 15 Division of Experimental Medicine, Department of Medicine, UCSF, San Francisco, USA.; 16 Department of Molecular and Cell Biology, University of California, Berkeley, USA.; 17 Department of Pathology, UCSF, San Francisco, USA.

**Keywords:** Development, Immunology, Metabolism, T cell development, Tolerance, Tregs

## Abstract

Neonatal life is marked by rapid antigen exposure, necessitating establishment of peripheral immune tolerance via conversion of naive CD4^+^ T cells into Tregs. We demonstrated heightened capacity for FOXP3 expression and tolerogenic function among cord blood versus adult blood naive CD4^+^ T cells. Further, this was linked to a distinct cord blood metabolic profile and elevated neonatal expression of the NADase, CD38. Early-life naive CD4^+^ T cells demonstrated a metabolic preference for glycolysis, which directly facilitated their differentiation trajectory. We revealed an age-dependent gradient in CD38 levels on naive CD4^+^ T cells and showed that high CD38 expression contributes to the glycolytic state and tolerogenic potential of neonatal CD4^+^ T cells, effects mediated at least partly via the NAD-dependent deacetylase SIRT1. Thus, the early-life window for peripheral tolerance in humans is critically enabled by the immunometabolic state of the naive CD4^+^ compartment.

## Abstract 2

Newborns’ immune systems are uniquely primed for tolerance. Naïve CD4+ T cells in cord blood have distinct metabolic traits enabling their regulatory potential during this critical early life window.

## Graphical abstract

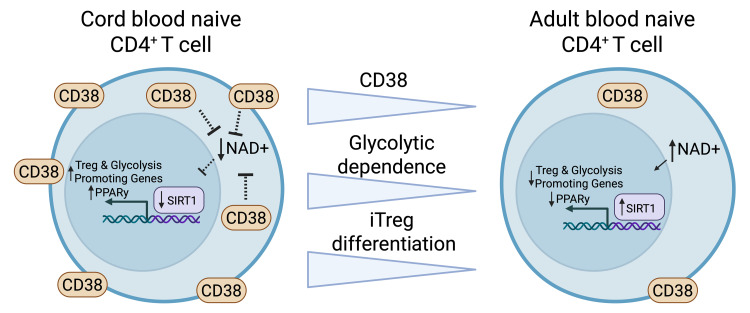

## Introduction

Neonatal life is marked by rapid exposure to new antigens, both peripheral tissue self-antigens and exogenous microbial and environmental stimuli. Developing immune tolerance to these antigens is critical to avoid inflammatory or allergic responses detrimental to homeostasis in developing tissues (1, 2). Tregs, expressing the transcription factor FOXP3, help establish and maintain this tolerance via expression of surface molecules, e.g., CTLA4, CD25, and CD73, and immunosuppressive cytokines, e.g., IL-10 and TGF-β. Notably, human fetal naive CD4^+^ T cells have heightened capacity to differentiate into Tregs compared with their adult counterparts (3). Prior work has revealed higher expression of Treg-associated genes in naive CD4^+^ T cells during human fetal development (4), with a progressive decline of this signature with age (5). Heightened postnatal Treg generation occurs in mice (6, 7). In humans, high Treg percentages in pediatric tissues (8) and capacity to limit food allergy via infant dietary exposure (9) also hint at an early-life window of tolerance. Prior work indicates that cord blood (CB) CD4^+^ T cells may have a higher propensity to upregulate Treg markers and manifest suppressive capacity versus their adult blood (AB) counterparts (10), but mechanisms supporting this remain ill-defined.

Metabolic pathways influence immune cell fate by modulating substrates needed for energy, protein, and nucleotide production and other cellular functions. Though most work has examined adult immune cells’ metabolic state, key overarching principles can inform hypotheses about early-life CD4^+^ T cell metabolism (11). Naive cells are relatively quiescent, their low ATP needs fulfilled primarily by mitochondrial oxidative phosphorylation (OXPHOS). Upon activation, heightened energy needs are met by rapidly increasing OXPHOS and glycolysis, but ATP dependency shifts toward glycolysis as a more immediate energy source. Finally, memory state transition pairs with reduced glycolysis and a shift back to OXPHOS (12). One study on metabolism of murine neonatal CD8^+^ T cells identified early-life preference for glycolysis facilitating effector differentiation while limiting memory generation (13). Analogous examination of neonatal naive CD4^+^ T cells is important given their multiple differentiation trajectories, i.e., Tregs, Th1, Th2, or Th17, and the long-term impact of these early cell fate decisions on adaptive immune function and tolerance.

CD38, or cyclic ADP ribose (cADPR) hydrolase, is a glycoprotein expressed by various cell types, including immune cells (14). It serves as a marker of early T cell activation (15) and CD8^+^ T cell exhaustion or metabolic dysfunction in chronic viral infections (16) or cancer (17) and as a proposed marker of human recent thymic emigrant (RTE) T cells (18). CD38 regulates NAD^+^ levels by catalyzing its conversion to ADP ribose and cADPR, depleting NAD^+^ levels (19). It also augments cellular calcium through cADPR (20) and facilitates tyrosine kinase activity downstream of TCR signaling (21). CD38 deletion (17) or pharmacological inhibition (14) has been linked to increased CD4^+^ T cell OXPHOS capacity, and transcriptional studies suggest association with a tolerogenic phenotype in murine Th17 cells (22). CD38’s enzymatic reduction of NAD^+^, i.e., NADase activity, may influence immune cell metabolism and function by altering NAD-dependent acetylases, such as sirtuin 1 (SIRT1), which affect mitochondrial biogenesis, autoimmunity, and FOXP3 degradation (23, 24), thus offering potential mechanisms by which CD38 could impact early-life immune cells.

Here, we aimed to define how the metabolic state of early-life human naive CD4^+^ T cells shapes their age-specific immune function. We find that the glycolytic preference of neonatal naive CD4^+^ T cells is intricately tied to high CD38 levels and that both these features support their enhanced capacity for FOXP3 expression and tolerogenic function. We further identify CD38’s NADase function and downstream effects on SIRT1 as key mediators. Collectively, these data offer potentially novel mechanistic insight into the early-life window for immune tolerance.

## Results

### CD38 is highly expressed on early-life naive CD4^+^ T cells.

**Figure 1:**
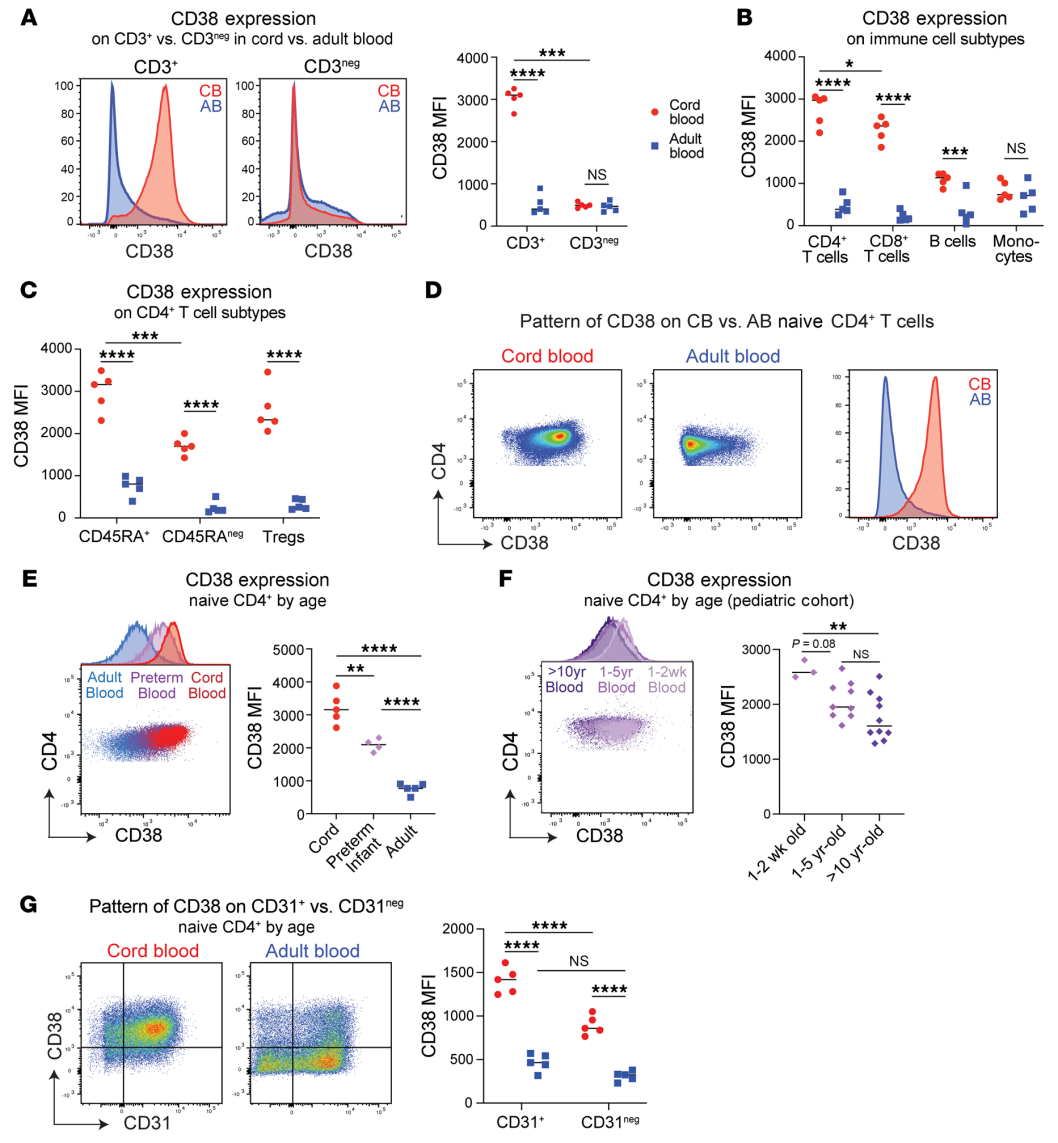
CD38 expression is highly enriched on naive early-life CD4^+^ T cells. (**A**) MFI of surface CD38 by flow cytometry on CD3^+^ and CD3^-^ cells from nonactivated peripheral CB and AB. CD38 MFI on (**B**) various immune cell populations and (**C**) CD4^+^ T cell subsets. (**D**) Pattern of CD38 surface staining on naive CD4^+^ T cells from CB and AB. (**E**) CD38 MFI on naive CD4^+^ T cells from CB, peripheral blood from 6-week-old preterm infants, or AB. (**F**) CD38 MFI on naive CD4^+^ T cells from peripheral blood from healthy pediatric donors. Data originally generated for van den Broek et al. (28). (**G**) CD38 and CD31 expression on nonactivated naive CD4^+^ T cells from CB and AB and the CD38 expression pattern on CD31^+^ versus CD31^–^ cells by age. (**B**–**D** and **G**) One of 2 representative experiments. (**E** and **F**) Performed once. Horizontal bars depict mean values in each graph. Two-way ANOVA with multiple comparisons used. *P ≤ 0.05, **P ≤ 0.02, ***P ≤ 0.001, ****P < 0.0001.

To investigate key features distinguishing the neonatal immune system, we performed flow cytometry on CB and AB PBMCs to measure key markers of cell identity and state. Extracellular CD38 expression was strikingly higher on CB T cells than AB (Figure 1A and Supplemental Figure 1A; supplemental material available online with this article; https://doi.org/10.1172/JCI200062DS1). Human CB contains CD38^+^ lymphoid progenitors (25, 26). However, further quantification of CD38 on different T cell subsets revealed high CD38 expression on CB CD4^+^ T cells, slightly less on CB CD8^+^ T cells, and even lower levels on B cells or monocytes (Figure 1B). CD38 expression was highest on CB naive versus non-naive CD4^+^ T cells, with CB naive CD4^+^ T cells exhibiting 4- to 5-fold more CD38 surface protein versus those in AB (Figure 1C).

Both CB and AB naive CD4^+^ T cells had a largely unimodal CD38 staining pattern, but as previously reported (18), AB contained a small population of cells with high CD38 expression (Figure 1D). Large age-dependent differences in CD38 were evident with combined intracellular and extracellular staining, confirming increased levels in CB reflected overall expression, not differential rates of internalization (Supplemental Figure 1B). As CD38 is often used as a T cell activation marker (14, 15), we measured its expression on naive CD4^+^ T cells after stimulating with human anti-CD3/CD28 antibody complexes. This confirmed an initial increase in CD38 expression after 3 hours and a smaller rise in AB (Supplemental Figure 1C). Of note, despite equivalent TCRβ expression (Supplemental Figure 1D), we observed higher Nur77 staining in CB versus AB 3 hours poststimulation (Supplemental Figure 1E), consistent with prior literature indicating higher TCR reactivity of early-life T cells (27). Age-dependent differences in CD38 expression were maintained through 48 hours of stimulation, after which expression in CB declined to AB levels (Supplemental Figure 1C).

To understand whether elevated CD38 expression reflects a unique CB state or a broader feature of the early-life window, we stained additional prenatal and postnatal samples. Naive CD4^+^ T cells from second trimester fetal spleen had even higher CD38 expression (Supplemental Figure 1F), whereas those from peripheral blood of 6-week-old preterm infants (corrected gestational age 0–1 weeks) showed intermediate CD38 levels relative to CB and AB (Figure 1E). Finally, reanalysis of flow cytometry data from a pediatric cohort (28) revealed progressive decline in mean CD38 expression on naive CD4^+^ T cells with increasing postnatal age (Figure 1F and Supplemental Figure 1G).

Based on the high rate of thymic output in early life, and recent work showing CD38 elevation among RTEs (18), we performed CD38 staining on CB and AB naive CD4^+^ T cells in parallel with the RTE marker, CD31 (29). CD38 expression was higher on CD31^+^ versus CD31^–^ cells in CB, with a nonsignificant trend toward higher CD38 on the CD31^+^ fraction in AB. However, large age-dependent differences in CD38 persisted among both CD31^+^ and CD31^–^ subsets (Figure 1G). Thus, while CD38 positively correlates with CD31 as a RTE marker, its higher expression on early-life naive CD4^+^ T cells does not seem a mere reflection of RTE frequency.

### Neonatal versus adult naive CD4^+^ T cells demonstrate a distinct transcriptional trajectory following stimulation.

To further elucidate key features distinguishing CB versus AB naive CD4^+^ T cells, we designed a longitudinal experiment to transcriptionally profile these cells at baseline and 3 time points after activation (Supplemental Figure 2A). Naive CD4^+^ T cells were sorted from CB or AB (Supplemental Figure 2B), then immediately processed (0 hour) or first stimulated in culture with anti-CD3/CD28 antibody complexes 3, 24, or 48 hours. Parallel analysis of analogously stimulated CB and AB naive CD4^+^ T cells did not reveal substantial cell death, based on viability dye or annexin 5 staining (Supplemental Figure 2, C and D). RNA-seq was performed on these bulk cell populations, generating a dataset that could be mined for both baseline transcriptional differences and distinct age-dependent trajectories after activation.

**Figure 2:**
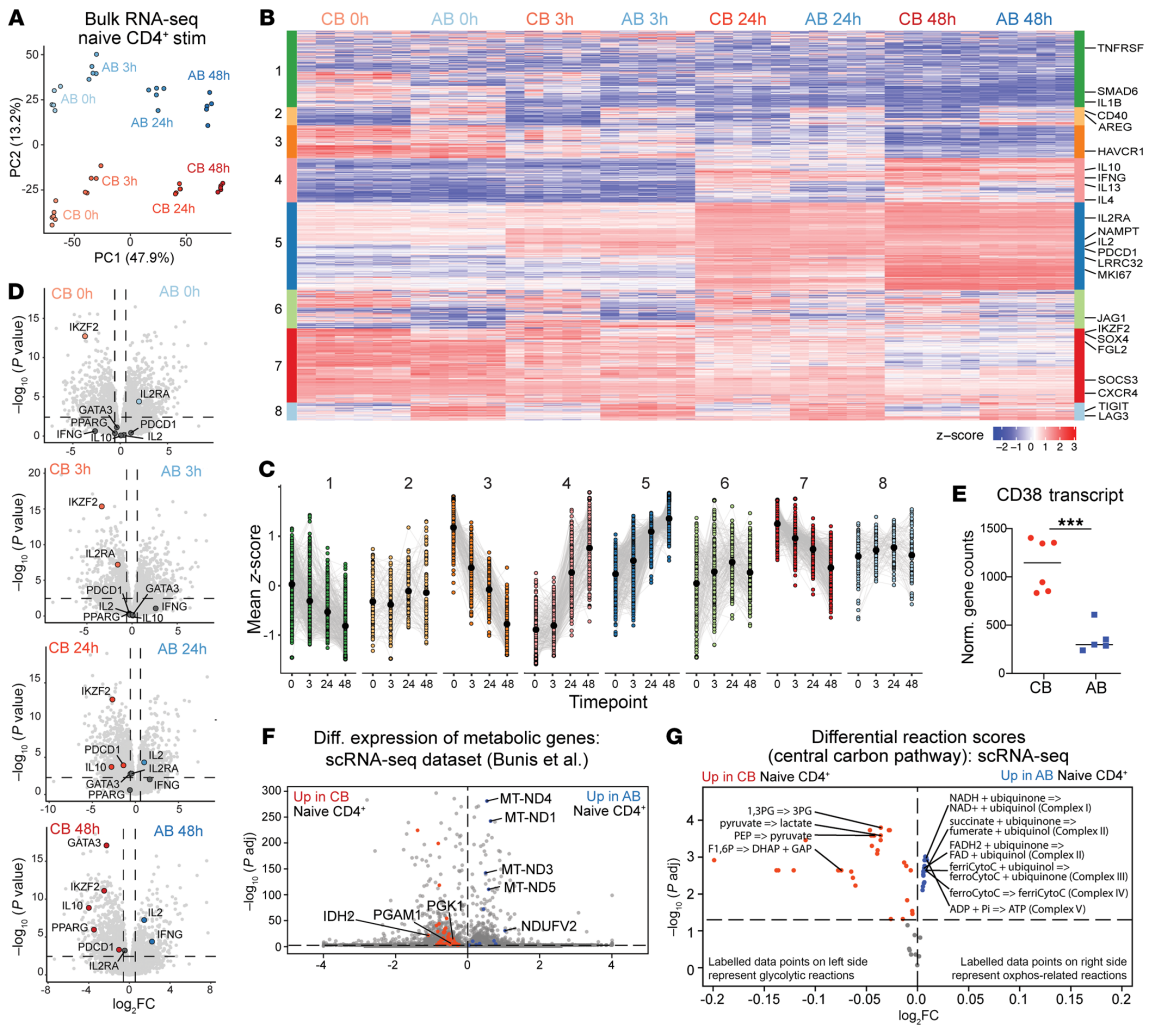
Transcriptomic data analysis and in silico modeling of CB versus AB naive CD4^+^ T cells reveal unique functional and metabolic programs. (**A**) PCA of bulk RNA-seq data from naive CD4^+^ T cells (CD4^+^CD8^neg^CD45RA^+^CD27^+^CCR7^+^CD95^neg^) sorted from CB and AB. Samples taken 0, 3, 24, and 48 hours after stimulation. (**B**) Heatmap of top variable features (i.e., gene clusters). (**C**) Mapping of cluster expression over time. Black dots show mean *z*-score of cluster genes per point. Colors correspond with clusters in **B**. (**D**) Volcano plots of differential gene expression for CB versus AB naive CD4^+^ T cells at each time point, with specific genes related to tolerance or activated effector cells highlighted. Horizontal dotted line: Benjamini-Hochberg–adjusted *P* of 0.05. (**E**) Normalized CD38 gene counts. (**F**) Differential expression of key metabolic genes in scRNA-seq dataset from Bunis et al. (5). (**G**) Compass comparison of CB versus AB naive CD4^+^ T cell metabolic states in central carbon metabolism, with glycolysis reactions as annotated in the Human1 GEM at left (up in CB) and downregulated OXPHOS reactions at right (up in AB). In **E**, horizontal bars depict mean values, with unpaired *t* test used and ***P ≤ 0.001.

Principal component analysis (PCA) was performed to visualize transcriptional differences between samples by time point and age (Figure 2A). PC1 (47.9% of variance) stratified samples longitudinally by time point, based on genes involved in cell proliferation and activation, e.g., *CDC25A*, *MCM10*, *MKI-67*, *IL2* (Supplemental Figure 2E). PC2 (13.2% of variance) separated CB from AB, with several axis-driving genes previously reported to be differentially expressed by age or associated with development, e.g., *SOX4* (30), *BCL11A* (31), *TSHZ2* (4) (Supplemental Figure 2, F and G). A heatmap of the most variable features revealed gene clusters with distinct patterns of expression (Figure 2B and Supplemental Tables 1–3). Some clusters encompassed genes that varied between CB and AB, largely independent of activation status (clusters 2, 6, 8), whereas others showed dynamic increases (clusters 4, 5) or decreases (clusters 3, 7) in expression poststimulation (Figure 2C). For example, cluster 4 contained several cytokine genes that were lowly expressed at baseline or 3 hours but later increased in AB or CB. Whereas AB naive CD4^+^ T cells upregulated *IFNG* and *IL2*, those from CB increased expression of *IL4*, *IL10*, and *IL13*. Key tolerance-related genes were enriched in CB versus AB at each time point. *IKZF2*, encoding Helios, was elevated in resting CB naive CD4^+^ T cells, as previously reported (5), whereas *IL2RA*, *IL10*, *GATA3*, *PPARG*, and *PDCD1* increased poststimulation (Figure 2D). Gene set enrichment analysis (GSEA) (32, 33) of ranked genes at baseline showed AB enrichment in host defense and cytokine responses, with more subtle enrichment of development-related pathways in CB (Supplemental Figure 2H). After 48 hours of stimulation, this developmental signature in CB was accompanied by additional processes such as regulation of cell activation and adhesion and proliferation, consistent with a slightly higher percentage of Ki-67^+^ cells in CB versus AB by flow cytometry at 24 and 48 hours (Supplemental Figure 2, I and J). Stimulated AB was enriched at 48 hours for terms such as defense against symbionts or virus, positive regulation of pattern recognition receptor signaling, and IL-17 production. Taken together, this analysis revealed a distinct transcriptional signature in CB cells at baseline and up to 48 hours after activation, with features suggesting a pro-tolerogenic signature.

### Neonatal naive CD4^+^ T cell transcriptome suggests a distinct metabolic profile.

Based on literature linking the metabolic state of neonatal naive CD8^+^ T cells to their differentiation trajectory and functional capacity (13, 34), we next investigated any baseline differences in metabolic state of CB and AB naive CD4^+^ T cells. Examining our RNA-seq dataset for differentially expressed metabolic genes revealed ~4-fold higher *CD38* transcript levels in CB versus AB naive CD4^+^ T cells, corroborating our flow cytometry data (Figure 2E and Supplemental Figure 2K). Certain glycolysis-related genes were also elevated in CB (Supplemental Figure 2L). Differential gene expression analysis on an independent single-cell RNA-sequencing (scRNA-seq) dataset of CB and AB naive CD4^+^ T cells (5) confirmed upregulation of glycolysis-related genes in CB, alongside downregulation of OXPHOS-critical mitochondrial genes (Figure 2F).

To further define the metabolic profile of CB naive CD4^+^ T cells, we turned to Compass, a flux balance analysis algorithm modeling metabolite flow through networks (22). Compass analysis of our baseline bulk RNA-seq data did not reveal statistically significant metabolic pathways in CB versus AB. Thus, we leveraged the same scRNA-seq dataset, performing Compass on the central carbon pathways (glycolysis, TCA, and OXPHOS). This showed striking upregulation of glycolysis reactions and downregulation of OXPHOS reactions in CB (Figure 2G). Thus, transcriptional signatures suggest that naive CD4^+^ T cells in CB have a distinct baseline metabolic state, with a possible preference for glycolysis.

### Neonatal naive CD4^+^ T cells demonstrate more tolerogenic potential upon stimulation than their adult counterparts.

**Figure 3:**
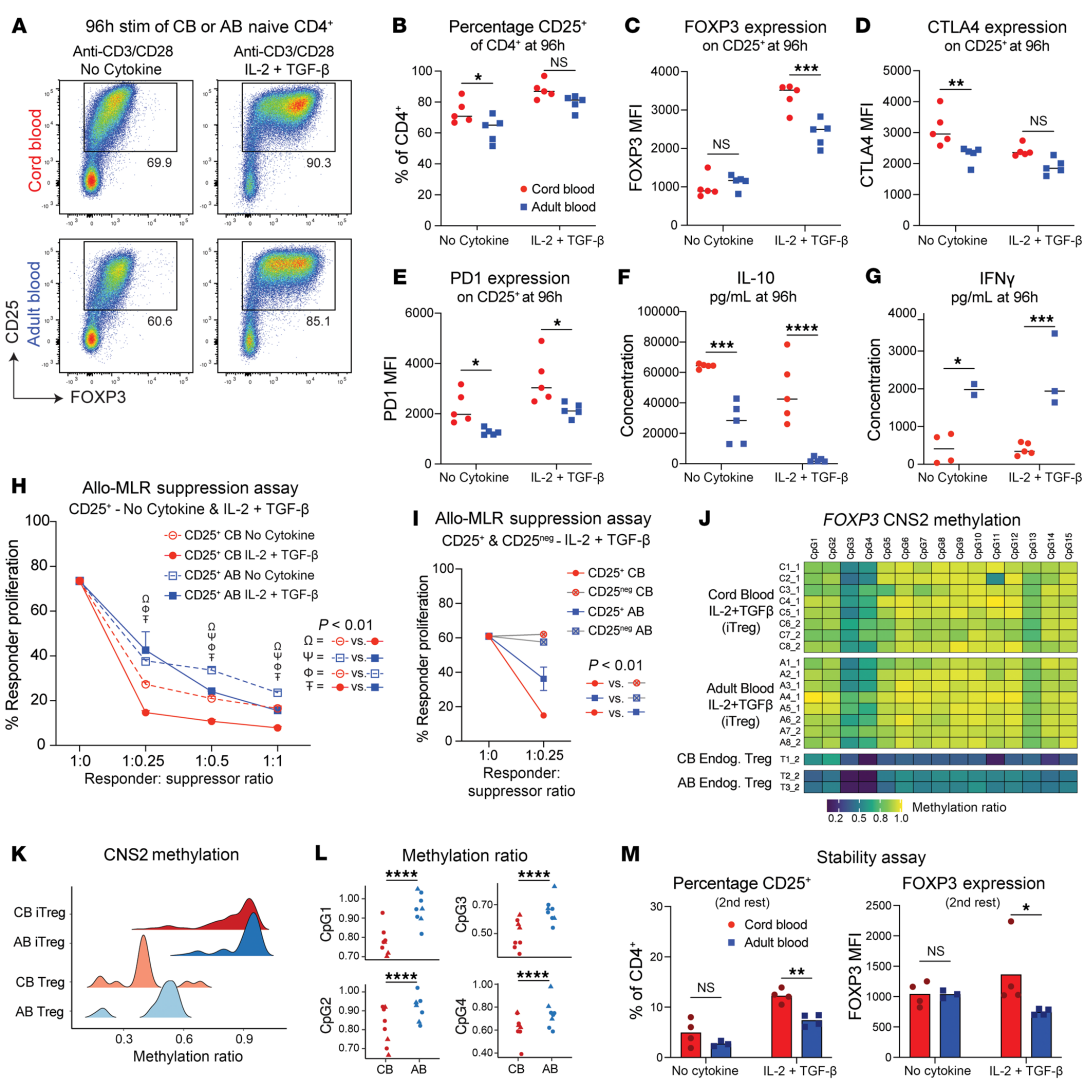
Neonatal naive CD4^+^ T cells demonstrate more tolerogenic potential upon stimulation than their adult counterparts. (**A** and **B**) CD25^+^ percentages from CB and AB naive CD4^+^ T cells after 96 hours of anti-CD3/CD28 stimulation under No Cytokine or IL-2 + TGF-β conditions. (**C**–**E**) Marker expression on CD25^+^ fraction by flow cytometry. (**F** and **G**) ELISA of cytokines in supernatant. (**H**) Allogeneic MLR measuring suppressive capacity of CB and AB No Cytokine and IL-2 + TGF-β-stimulated cells. (**I**) Allogeneic MLR assay for CD25^+^ versus CD25^neg^ iTreg-stimulated cells. (**J**–**L**) *FOXP3* CNS2 methylation was assessed by bisulfide sequencing in CD4^+^CD25^+^CD127^lo^ cells from iTreg cultures and endogenous blood Tregs (CD4^+^FOXP3^+^CD25^+^CD127^lo^). Two separate experiments were analyzed together with adjustment for batch effects. (**J**) Heatmap, (**K**) violin plot, and (**L**) graphs of CpG island methylation ratios. (**M**) FOXP3 stability assessed through 2 rounds of stimulation and rest. CD25^+^ and FOXP3 MFI on CD25^+^ cells after second rest. Data points represent individual donors. (**H** and **I**) *N* = 5/group. (**A**–**E**) One of 3 representative experiments. (**H**) One of 2 representative experiments. (**F**, **G**, **I**, and **M**) Performed once. (**J**–**L**) Combined data from 2 experiments. (**B**–**G** and **M**) Two-way ANOVA with multiple comparisons used. (**I**) One-way ANOVA with multiple comparisons used. (**L**) Linear mixed model (included batch correction). *P ≤ 0.05, **P ≤ 0.02, ***P ≤ 0.001, ****P < 0.0001. Horizontal bars and column heights in panels **B**–**G** and **M** depict mean values. In **H** and **I**, mean value plus standard deviation is shown.

We interrogated the functional relevance of increased tolerance-related gene expression among stimulated CB naive CD4^+^ T cells. A naive CD4^+^ T cell negative selection kit was used to isolate CD4^+^CD45RA^+^ T cells that were CD25^–^ and FOXP3^–^ (Supplemental Figure 3, A and B). These cells were then cultured with human anti-CD3/CD28 soluble antibodies without added cytokines, replicating our RNA-seq conditions (“No Cytokine” condition). Separately, we performed analogous stimulation in the presence of IL-2 and TGF-β, replicating published conditions for “induced Treg” generation (4) (“iTreg” or “IL-2+TGF-β” condition). Flow cytometry at 96 hours revealed that stimulated CB and AB CD4^+^ T cells demonstrated distinct patterns of FOXP3 and CD25 expression (Figure 3A), despite equivalent markers of recent activation and proliferation (Supplemental Figure 3, C and D). Under No Cytokine conditions, a higher percentage of CB cells became CD25^+^ (Figure 3B). Irrespective of age, the majority of these CD25^+^ cells expressed FOXP3 at levels approaching that of endogenous blood Tregs (Figure 3C and Supplemental Figure 3E). This heightened frequency of CD25^+^ cells from CB was replicated under No Cytokine conditions using various methods of CD3/CD28 stimulation (Supplemental Figure 3F). In the presence of IL-2 and TGF-β, the percentage of CD25^+^ cells (i.e., iTregs) again trended higher in CB (Figure 3B). As expected from iTreg culture conditions, these cells expressed much higher levels of FOXP3. Yet, iTregs generated from CB had even higher FOXP3 levels than their AB counterparts (Figure 3, A and C).

Though similar CD25 levels were seen irrespective of age or culture condition, CD25^+^ cells generated from CB showed higher expression of several Treg effector molecules, including CTLA4, PD-1, Helios, and ICOS (Figure 3, D and E, and Supplemental Figure 3, G–I). ELISA on CB culture supernatants at 96 hours also revealed increased levels of IL-10 (Figure 3F). AB supernatant contained more IFN-γ under either condition (Figure 3G) and more IL-2 under No Cytokine conditions (Supplemental Figure 3J). Aside from slightly more IL-4 in No Cytokine AB supernatant, no significant age-dependent differences were seen for IL-4, IL-5, or IL-13 (Supplemental Figure 3, K–M).

We next assessed the relative suppressive capacity of CD25^+^ cells derived from CB or AB in an allogeneic mixed lymphocyte reaction (MLR). Live CD4^+^CD25^+^CD127^lo^ “Suppressor” cells were sorted from No Cytokine or iTreg cultures at 96 hours and cocultured at varying ratios with cell trace violet–labeled PBMC “Responder” cells from an unrelated adult donor and irradiated PBMCs to facilitate antigen presentation from a second unrelated adult donor (Supplemental Figure 3N). Responder cell proliferation after 7 days revealed significant differences in suppression, based on both age and culture conditions used to generate the Suppressor cells (Supplemental Figure 3O). As anticipated, iTreg conditions led to more potently suppressive cells versus No Cytokine stimulation, irrespective of age. Intriguingly, CB-derived Suppressor cells from either culture condition were more potent than their AB-derived counterparts, demonstrating higher dose-dependent suppression at all 3 ratios tested (Figure 3H). A repeat of this MLR assay at the lowest suppressor-to-responder ratio confirmed the higher potency of CB versus AB Suppressor cells generated under iTreg conditions and showed this tolerogenic capacity was restricted to CD25^+^ cells (Figure 3I). Thus, iTreg cells from CB display more suppressive capacity, especially those generated in the presence of IL-2 and TGF-β.

Treg stability and function has been linked to demethylation of conserved noncoding sequence 2 (CNS2), a CpG-rich *FOXP3* intronic *cis-*regulatory element (35). We thus performed methylation analysis of this locus in sorted CD25^+^ cells generated from CB or AB iTreg cultures. Endogenous Tregs sorted from AB and CB (CD4^+^FOXP3^+^CD25^+^CD127^lo^) served as positive controls. Consistent with previous studies and known limitations of iTregs generated under these culture conditions (36), CD25^+^ cells from stimulated CB or AB cultures showed substantially more methylation than endogenous Tregs (Figure 3, J and K). Nonetheless, subtle but statistically significant differences were seen among CD25^+^ cells from CB versus AB, specifically at CpG sites 1–4 and 11 (Figure 3L and Supplemental Figure 3P).

Finally, we sought to assess if CNS2 demethylation status corresponded with increased stability of FOXP3 expression among CD25^+^ cells generated from CB versus AB. We adopted previously published methods for testing FOXP3 stability, in which cells are subjected to 2 sequential rounds of stimulation and rest (37) (Supplemental Figure 3Q). At the “first rest” time point, percentages of CD25^+^ cells and FOXP3 MFIs had dropped versus their baseline benchmarks (see Figure 3, B and C), without observed differences based on age or culture condition used for initial stimulation (Supplemental Figure 3R). However, at the “second rest” time point, CB naive CD4^+^ T cells initially subjected to iTreg conditions demonstrated significantly higher percentages of CD25^+^ cells, which also expressed more FOXP3, versus their AB counterparts (Figure 3M). Thus, following culture in iTreg conditions, naive CD4^+^ T cells from CB can become more functionally suppressive than their AB counterparts and demonstrate comparatively more CNS2 demethylation and more stable FOXP3 expression, albeit still much less than seen in endogenous Tregs (36).

### Neonatal naive CD4^+^ T cells express distinct metabolic molecules versus their adult counterparts.

**Figure 4:**
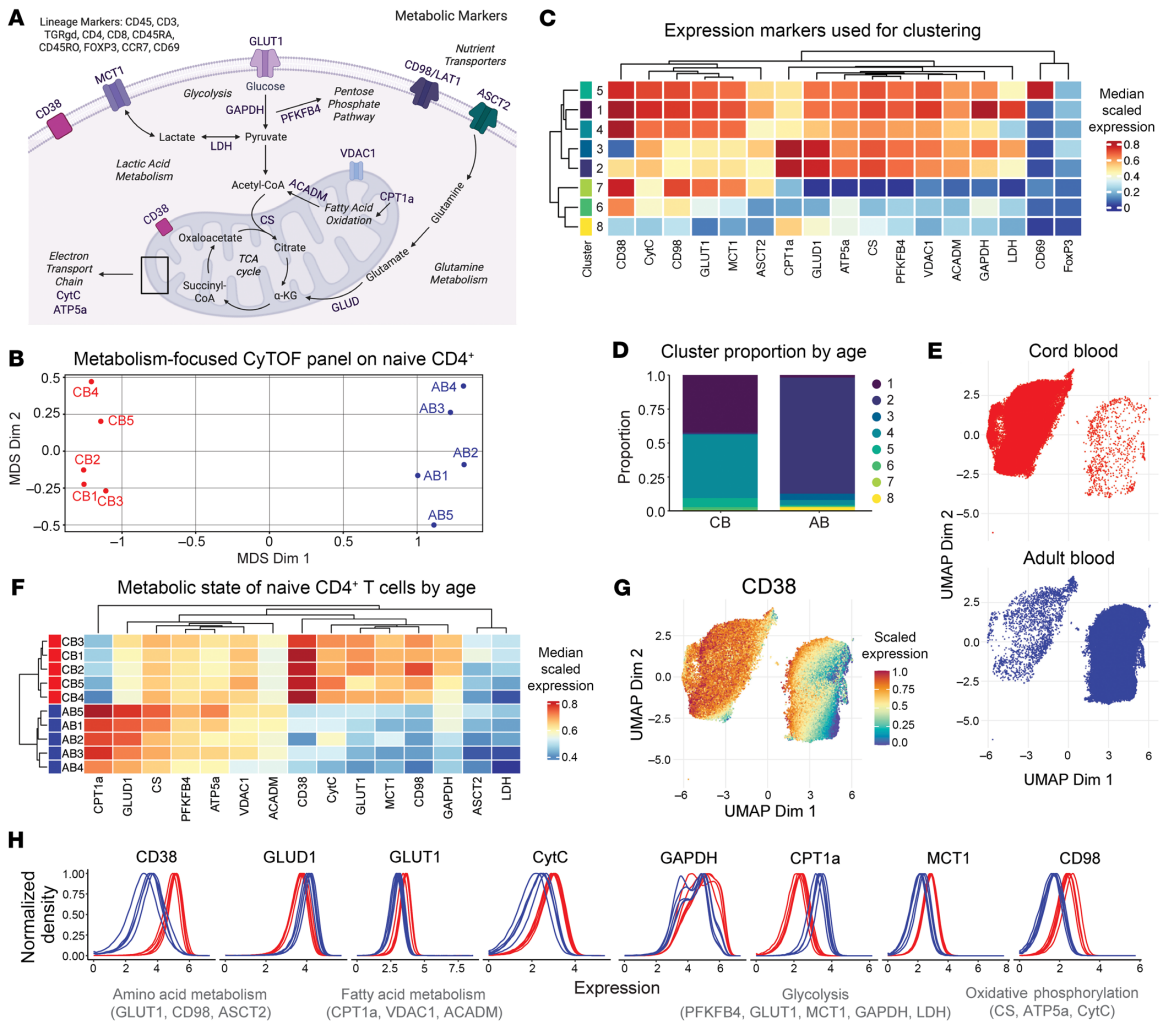
Neonatal naive CD4^+^ T cells express distinct patterns of metabolic proteins versus their adult counterparts. (**A**) Schematic of CyTOF metabolic markers in corresponding pathways. (**B**) MDS of median marker expression to examine relatedness of CB and AB naive CD4^+^ T cell metabolic state in an unsupervised manner. (**C**) Median scaled expression of metabolic and lineage markers by cluster, (**D**) abundance of clusters by age, (**E**) uniform manifold approximation and projection (UMAP) distribution of CB and AB naive CD4^+^ T cells. (**F**) Expression of metabolic proteins across individual CB and AB samples. (**G**) Intensity plot of CD38 expression. (**H**) Expression patterns of metabolic markers on individual CB and AB donors.

Based on links between the metabolic state of neonatal murine naive CD8^+^ T cells and their distinct differentiation trajectory (13), we wondered if metabolic features of CB naive CD4^+^ T cells promote their capacity to develop tolerogenic properties. Expression of nutrient transporters, transcription factors, and metabolic enzymes measured via mass cytometry (CyTOF) strongly correlate with T cell metabolic state (38). Thus, we developed an immunometabolism-focused CyTOF panel (Figure 4A) and performed analysis of naive CD4^+^ T cells (CD45RA^+^CCR7^+^) from CB and AB (Figure 2, G and H, and Supplemental Figure 2H). Using multidimensional scaling (MDS) of median marker expression to examine the metabolic state of CB and AB naive CD4^+^ T cells in an unsupervised manner, we observed distinct patterns of expression among CB and AB naive CD4^+^ T cells (Figure 4B). To examine the different metabolic states present in the naive CD4^+^ T cell pool across age, we performed clustering with FlowSOM (39), which yielded 8 distinct clusters that were largely nonoverlapping by age (Figure 4, C–E and Supplemental Figure 4A).

Consistent with our RNA-seq data, CB naive CD4^+^ T cells showed increased expression of proteins central to glycolysis and decreased expression of molecules associated with fatty acid oxidation (FAO) (Figure 4F). Differential abundance analysis (40) showed CB naive CD4^+^ T cells to be significantly enriched in clusters 1, 4, and 5 (Figure 4D), which expressed proteins linked to glycolysis and lactate metabolism, i.e., a glucose transporter (GLUT1), GAPDH, lactate dehydrogenase (LDH), and monocarboxylate transporter 1 (MCT1) (Figure 4F) (41). Mass cytometry also recapitulated the age-dependent CD38 gradient observed previously via flow cytometry and RNA-seq (Figure 4G and Supplemental Figure 4B).

Compared with CB naive CD4^+^ T cells, AB cells appeared more reliant on FAO and OXPHOS, as indicated by higher levels of carnitine palmitoyltransferase (CPT1a), an essential enzyme of FAO, and lower levels of glycolysis-associated proteins (Figure 4, F and H, and Supplemental Figure 4C). AB cells also exhibited a more homogenous metabolic phenotype, with the majority falling into the CPT1a-high cluster 2 (Figure 4, C and D). Although differential expression of proteins associated with glycolysis, lactic acid metabolism, FAO, and OXPHOS drove the major age-related differences in metabolic phenotypes, these data also hinted at subtler differences. For example, CB expressed more CD98, which is involved in amino acid transport, whereas AB expressed more glutamate dehydrogenase (GLUD1), a key enzyme in glutaminolysis (Figure 4, F and H). Together, these data further indicate that naive CD4^+^ T cells from CB are metabolically unique and exhibit features of glycolytic dependence.

### Neonatal naive CD4^+^ T cells are more reliant on glycolysis than their adult counterparts.

**Figure 5:**
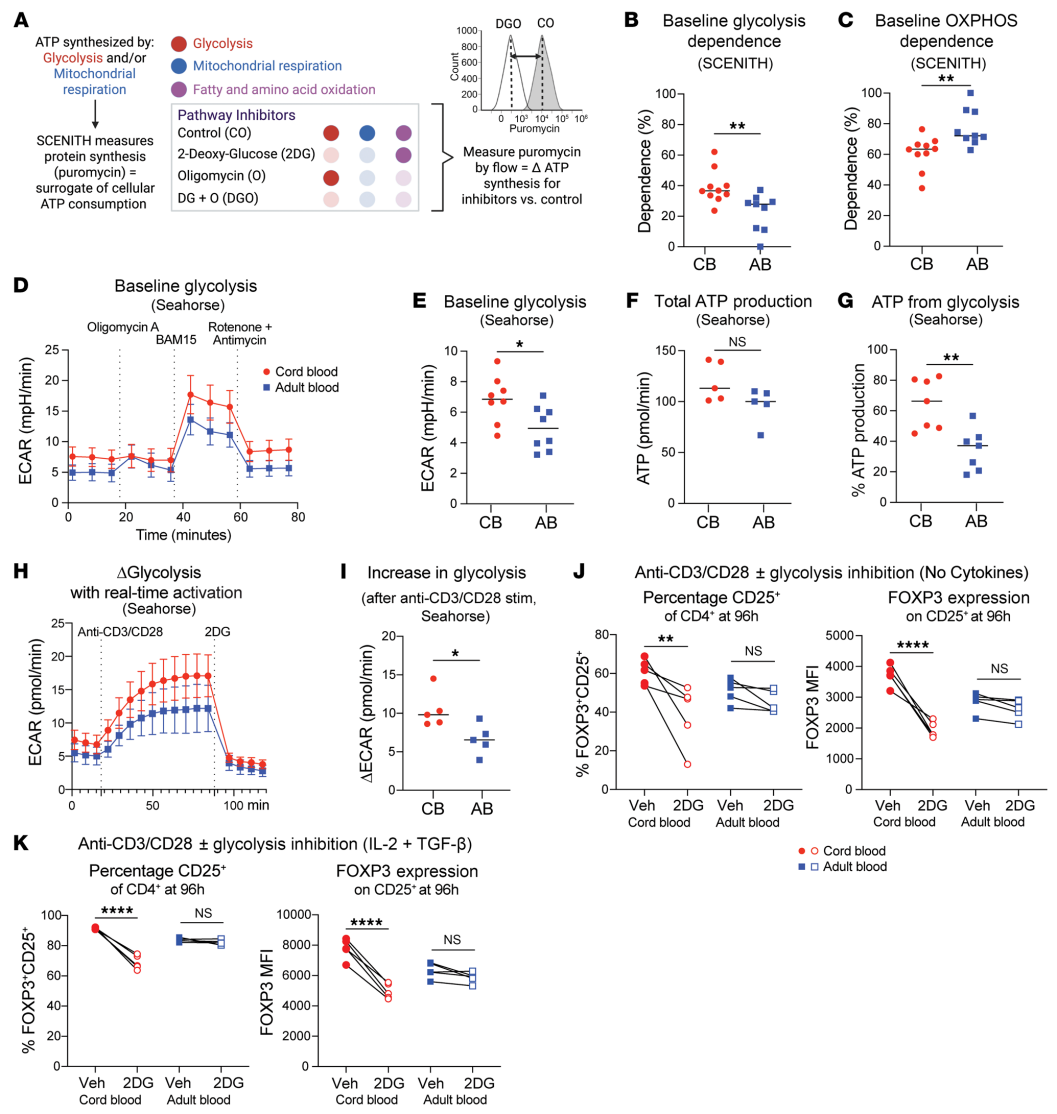
Neonatal naive CD4^+^ T cells demonstrate a metabolic preference for glycolysis. (**A**) SCENITH workflow. (**B** and **C**) Baseline dependence on glycolysis and mitochondrial OXPHOS in nonactivated CB and AB naive CD4^+^ T cells measured via SCENITH. (**D**) Seahorse ECAR plot of nonactivated CB and AB naive CD4^+^ T cells. Baseline ECAR (**E**), total ATP from glycolysis (**F**), percent ATP from glycolysis (**G**) between CB and AB naive CD4^+^ T cells. (**H** and **I**) Change in ECAR for CB and AB naive CD4^+^ T cells after acute TCR activation. (**J** and **K**) Percentage of CD25^+^ cells and FOXP3 MFI from CB and AB naive CD4^+^ after No Cytokine or IL-2+TGF-β stimulation with/without concurrent glycolysis inhibition via 2DG. Vehicle = DMSO. Data points represent individual donors. **B**, **C**, and **E** are 2 combined experiments. (**D** and **F**–**K**) One of 2 representative experiments. Unpaired *t* test used for **B**–**I** and paired *t* test for **J** and **K**. *P ≤ 0.05, **P ≤ 0.02, ****P < 0.0001. **B**, **C**, **E**–**G** and **I** depict mean values. In **D** and **H**, mean value plus standard deviation is shown.

We next used SCENITH (42), a flow cytometry–based method that combines pharmacologic inhibition of metabolic pathways with puromycin antibody-based readout of cellular ATP production (Figure 5A) to functionally test the metabolic dependencies of neonatal versus adult naive CD4^+^ T cells. SCENITH can measure both the pathway by which cells generate energy (i.e., glycolysis versus OXPHOS) and their preferred energy source (i.e., glucose versus fatty acid and amino acid oxidation). Pilot experiments on naive CD4^+^ T cells before and after cryopreservation did not reveal significant metabolic differences by SCENITH (Supplemental Figure 5A). Thus, we opted to perform our metabolism-focused experiments on cryopreserved samples to enable parallel processing and analysis of multiple donors.

SCENITH analysis of resting (baseline) CB and AB naive CD4^+^ T cells showed equivalent puromycin staining between groups in the absence of metabolic inhibition, suggesting relatively equivalent ATP use irrespective of age (Supplemental Figure 5, B and C). Changes to puromycin incorporation in the setting of various pathway inhibitors revealed CB naive CD4^+^ T cells had increased relative dependence on glycolysis versus AB, with a converse decrease in OXPHOS (Figure 5, B and C). We did not observe distinct energy substrate preferences by age, with CB and AB cells demonstrating higher reliance on fatty acid and amino acid oxidation versus glucose, as expected for nonactivated, resting cells (Supplemental Figure 5D).

To validate our SCENITH results and measure absolute energy use, we performed Seahorse assay on resting CB and AB naive CD4^+^ T cells. CB showed higher baseline extracellular acidification rate (ECAR), reflective of increased glycolysis (Figure 5, D and E). While Seahorse did not show age-dependent differences in total ATP generation, CB naive CD4^+^ T cells did show higher ATP from glycolysis (Figure 5, F and G). No differences were seen in baseline oxygen consumption rate (OCR; measurement of OXPHOS), ATP from OXPHOS, or spare respiratory capacity between ages (Supplemental Figure 5, E–H). We measured ECAR and OCR continuously following anti-CD3/CD28 activation. This showed CB naive CD4^+^ T cells increased their glycolytic activity more than AB following TCR stimulation, which was matched by age-specific OXPHOS increases (Figure 5, H and I, and Supplemental Figure 5, I and J).

We next probed whether the glycolytic state of CB naive CD4^+^ T cells was linked to their capacity for FOXP3 expression upon stimulation. We titrated 2-deoxy-d-glucose (2DG), a pharmacological inhibitor of glycolysis, to a dose that did not impact cell viability, proliferation, or activation during 96-hour stimulation (Supplemental Figure 5, K–M). We then treated CB and AB naive CD4^+^ T cells with 2DG in tandem with No Cytokine or iTreg culture conditions. 2DG led to a significant reduction in the percentage of CD25^+^ cells from CB, as well as their FOXP3 expression, under either culture condition, without analogous effects on AB (Figure 5, J and K). Together, these data confirm higher baseline glycolytic dependency among CB versus AB naive CD4^+^ T cells connected with their heightened tolerogenic potential.

### CD38 inhibition limits glycolysis and FOXP3 expression by CB naive CD4^+^ T cells.

**Figure 6:**
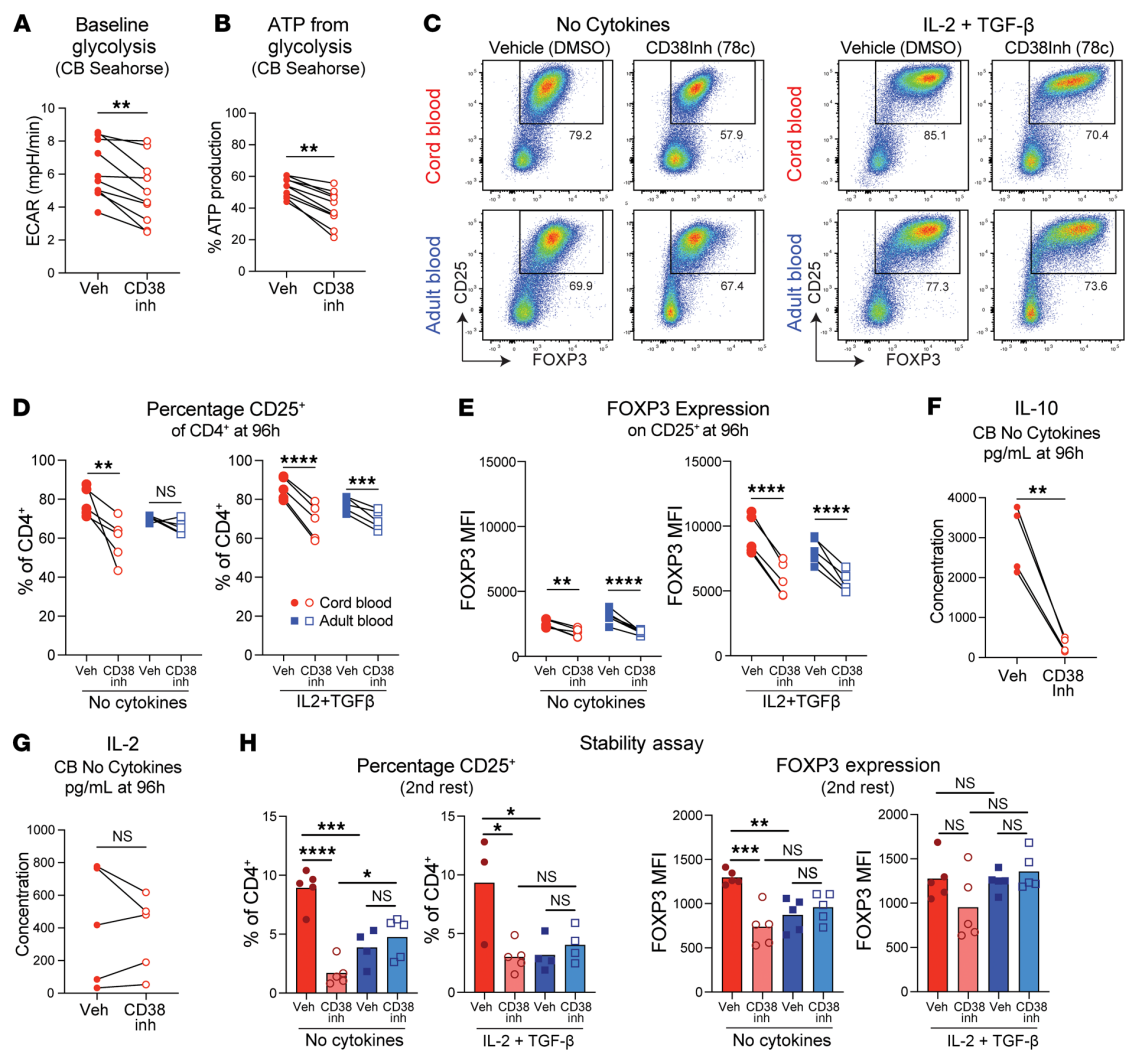
Inhibition of CD38 activity in cord blood naive CD4^+^ T cells reduces their FOXP3 expression and glycolytic capacity. (**A**) ECAR and (**B**) percent ATP from glycolysis in vehicle (DMSO) and CD38 inhibitor (5 μM 78c) CB naive CD4^+^ T cells. (**C**–**E**) Representative flow plots and graphs of percentage CD25^+^ cells and their FOXP3 MFI after 96 hours of stimulation under No Cytokine or IL-2+TGF-β conditions, where cells were treated with DMSO or 78c before and during activation. (**F** and **G**) ELISA-based measurement of cytokines in culture supernatant of DMSO- or 78c-treated CB No Cytokine cultures. (**H**) Treg stability assay following No Cytokine or IL-2+TGF-β culture with 78c or DMSO added before and during initial activation. Percentage CD25^+^ cells and their FOXP3 MFI for each condition. Data points represent individual donors. (**A**–**E**) One representative experiment from ≥ 2 repeats. (**F**–**H**) Performed once. Paired *t* tests used for **A**, **B**, and **D**–**G** and 2-way ANOVA with multiple comparisons used for **H**. *P ≤ 0.05, **P ≤ 0.02, ***P ≤ 0.001, ****P < 0.0001. Column heights in **H** depict mean value.

To dissect functional links between CD38 expression, glycolytic dependency, and FOXP3 expression, we took advantage of the potent and specific CD38 pharmacological inhibitor, 78c (14). Incubation of CB naive CD4^+^ T cells with 78c for 24 hours led to reduced ECAR, decreased ATP from glycolysis (Figure 6, A and B), and increased OXPHOS activity as measured by Seahorse (Supplemental Figure 6A). Analogous treatment of AB naive CD4^+^ T cells did not alter these metabolic parameters (Supplemental Figure 6B). Additional titration of 78c followed by SCENITH confirmed a dose-dependent decrease in CB glycolysis with progressive CD38 inhibition, without effects on AB (Supplemental Figure 6C).

We extended 78c treatment through 96 hours of stimulation with or without IL-2 and TGF-β. CD38 inhibition significantly decreased percentages of CD25^+^ cells from CB but not AB under No Cytokine conditions, whereas this population decreased with 78c treatment of CB and AB iTreg cultures. FOXP3 level on CD25^+^ cells was blunted by CD38 inhibition irrespective of age or culture condition (Figure 6, C–E). 78c treatment did not lower CD69 expression (Supplemental Figure 6D) but lowered Ki-67 expression across all groups (Supplemental Figure 6E), an effect not observed among CD25^+^ cells specifically (Supplemental Figure 6F). Under No Cytokine conditions, CB cells displayed greater sensitivity to 78c compared with AB cells, with lower doses reducing CD25^+^ cell percentages and FOXP3 expression in CB, whereas viability was unaffected even at high 78c concentrations and dose-dependent effects on Ki-67 were similar across age (Supplemental Figure 6G).

ELISA on supernatants from No Cytokine cultures revealed a decrease in IL-10 but not IL-2 in 78c-treated CB (Figure 6, F and G). FOXP3 stability assays comparing AB and CB cells initially cultured with or without 78c showed no significant effects at the first rest (Supplemental Figure 6H), but significant reductions in both CD25^+^ percentages and FOXP3 MFI were seen in 78c-treated No Cytokine CB cultures after the second rest. Under iTreg conditions, CD38 inhibition likewise reduced CB CD25^+^ cells, with a downward trend in their FOXP3 expression. AB cells showed no effects from 78c under either culture condition (Figure 6H).

To explore if CB cells are uniformly more sensitive to CD38 inhibition, we tested the effect of 78c on Th2 differentiation, a process in which glycolysis has been specifically implicated (43). Naive CD4^+^ T cells were stimulated via plate-coated anti-CD3/CD28 antibodies plus IL-4 and anti–IFN-γ for 3 days, then rested overnight with IL-2, IL-4, and anti–IFN-γ before PMA/ionomycin restimulation and flow cytometry. Equivalent percentages of IL-4^+^IL-13^+^ cells were seen in CB and AB without 78c, and CD38 inhibition led to equivalent reductions in this population irrespective of age (Supplemental Figure 6, I and J). Thus, while CD38 inhibition can impact naive CD4^+^ differentiation down a Th2 trajectory, heightened CB sensitivity to CD38 inhibition is not seen in this setting as for FOXP3 expression.

### The CD38/NAD/SIRT1 axis modulates FOXP3 expression in stimulated neonatal naive CD4^+^ T cells.

**Figure 7:**
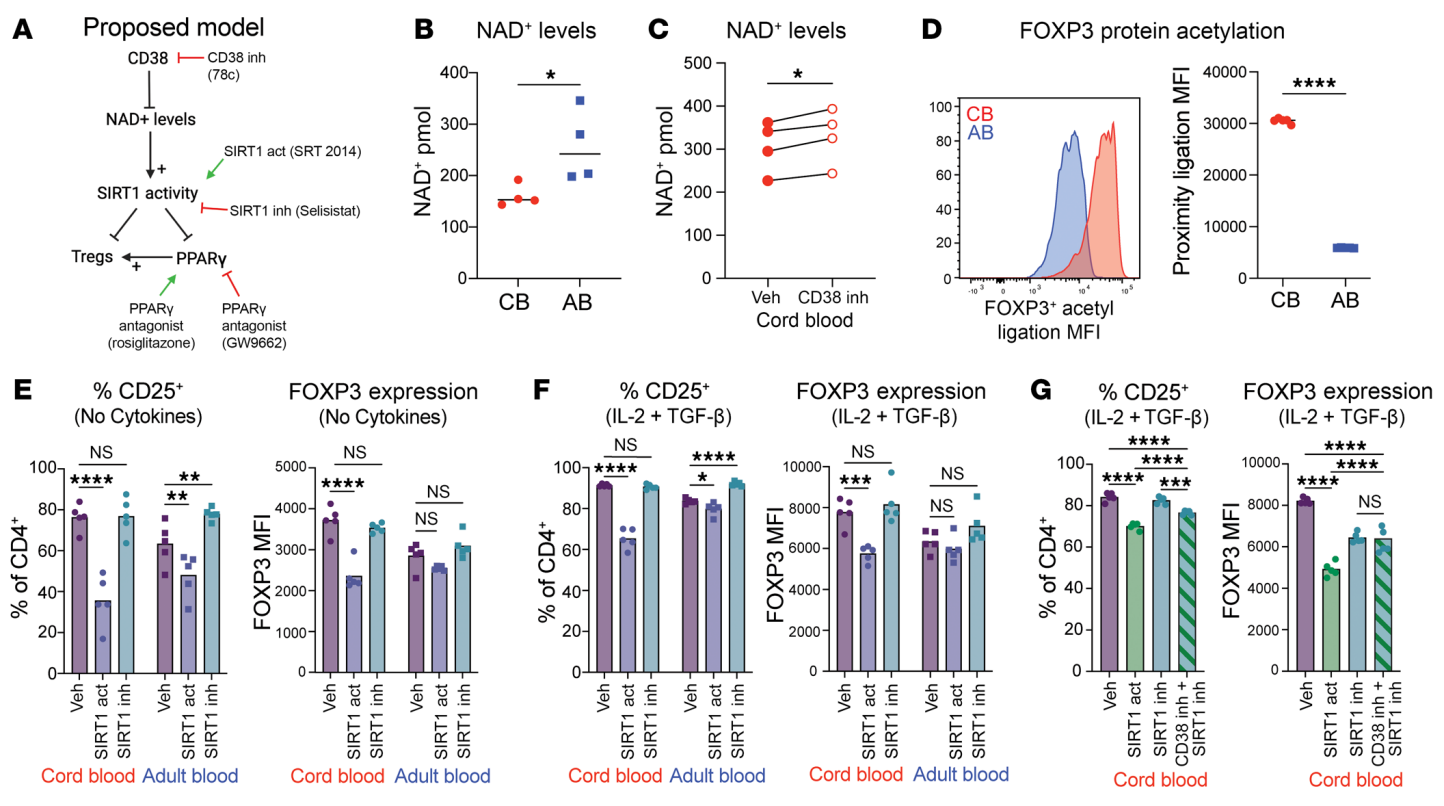
The NAD^+^-dependent deacetylase, SIRT1, modulates FOXP3 expression in stimulated neonatal naive CD4^+^ T cells. (**A**) Working model of CD38-NAD-SIRT1-Tregs-PPARγ interactions with pharmacological perturbations noted. (**B** and **C**) NAD^+^ levels in CB and AB naive CD4^+^ T cells (**B**) and DMSO- or 78c-treated CB naive CD4^+^ T cells (**C**). (**D**) FOXP3 acetylation in CB and AB naive CD4^+^ T cells via proximity ligation assay (PLA). (**E**–**G**) CD25^+^ percentage and FOXP3 MFI among CB and AB naive CD4^+^ T cells treated with DMSO (vehicle), SIRT1 activator, or SIRT1 inhibitor (**E** and **F**) or vehicle, CD38 inhibitor, SIRT1 inhibitor, or both (**G**) before activation under No Cytokine or IL-2+TGF-β conditions. Data points represent individual donors. **B**–**F** show 1 of 2 experimental replicates. **G** was performed once. Unpaired *t* test was used for **B** and **D** and paired *t* test for **C**. Two-way ANOVA with multiple comparisons used for **E**–**G**. *P ≤ 0.05, **P ≤ 0.02, ***P ≤ 0.001, ****P < 0.0001. Horizontal bars in **B** and **D** and column heights in **E**–**G** depict mean values.

CD38-mediated synthesis of ADP ribose and cADPR depletes cellular NAD^+^ (14), which serves a cofactor for oxidoreductases, an electron donor for redox reactions, and a substrate for the sirtuin family of deacetylases (44). We were particularly intrigued how this last NAD^+^ function might shape the metabolic state and tolerogenic potential of CB naive CD4^+^ T cells, as SIRT1 has been inversely correlated with glycolytic function (45) and can target FOXP3 protein for degradation (23, 24). A proposed model (Figure 7A) was supported by lower NAD^+^ levels in CB compared with AB naive CD4^+^ T cells (Figure 7B), which recovered with pharmacological CD38 inhibition (Figure 7C). No age-related differences were seen in global H3K27ac levels, a histone modification associated with heightened *FOXP3* induction (46) (Supplemental Figure 7A), but flow-based proximity ligation assays revealed significantly higher levels of acetylated FOXP3 protein in CB versus AB (Figure 7D). This supports a mechanistic model in which reduced SIRT1 activity in CB naive CD4^+^ T cells results in less FOXP3 deacetylation, leading to higher FOXP3 levels due to decreased degradation.

To assess SIRT1’s role in FOXP3 regulation in stimulated naive CD4^+^ T cells, we treated CB and AB cells with a SIRT1 activator (SRT 2104), SIRT1 inhibitor (selisistat), or DMSO for 24 hours before activation under No Cytokine or iTreg conditions. After 96 hours, SIRT1 activation reduced CD25^+^ percentages and FOXP3 levels in CB, with a modest effect on CD25^+^ cells in AB and no significant impact on FOXP3 expression. SIRT1 inhibition had no impact on FOXP3 in CB but modestly increased percentages of CD25^+^ cells in AB (Figure 7, E and F, and Supplemental Figure 7B). Combining CD38 and SIRT1 inhibition during CB activation under iTreg conditions partially restored CD25^+^ percentages and FOXP3 levels compared with CD38 inhibition alone (Figure 7G). SCENITH analysis showed SIRT1 activation decreased glycolytic capacity and increased OXPHOS capacity in resting CB naive CD4^+^ T cells (Supplemental Figure 7C). These findings suggest that low SIRT1 activity in CB enhances FOXP3 expression during stimulation, partially through metabolic effects, while higher baseline SIRT1 activity in AB restricts FOXP3 expression but can be pharmacologically modulated to adjust tolerogenic potential.

**Figure 8:**
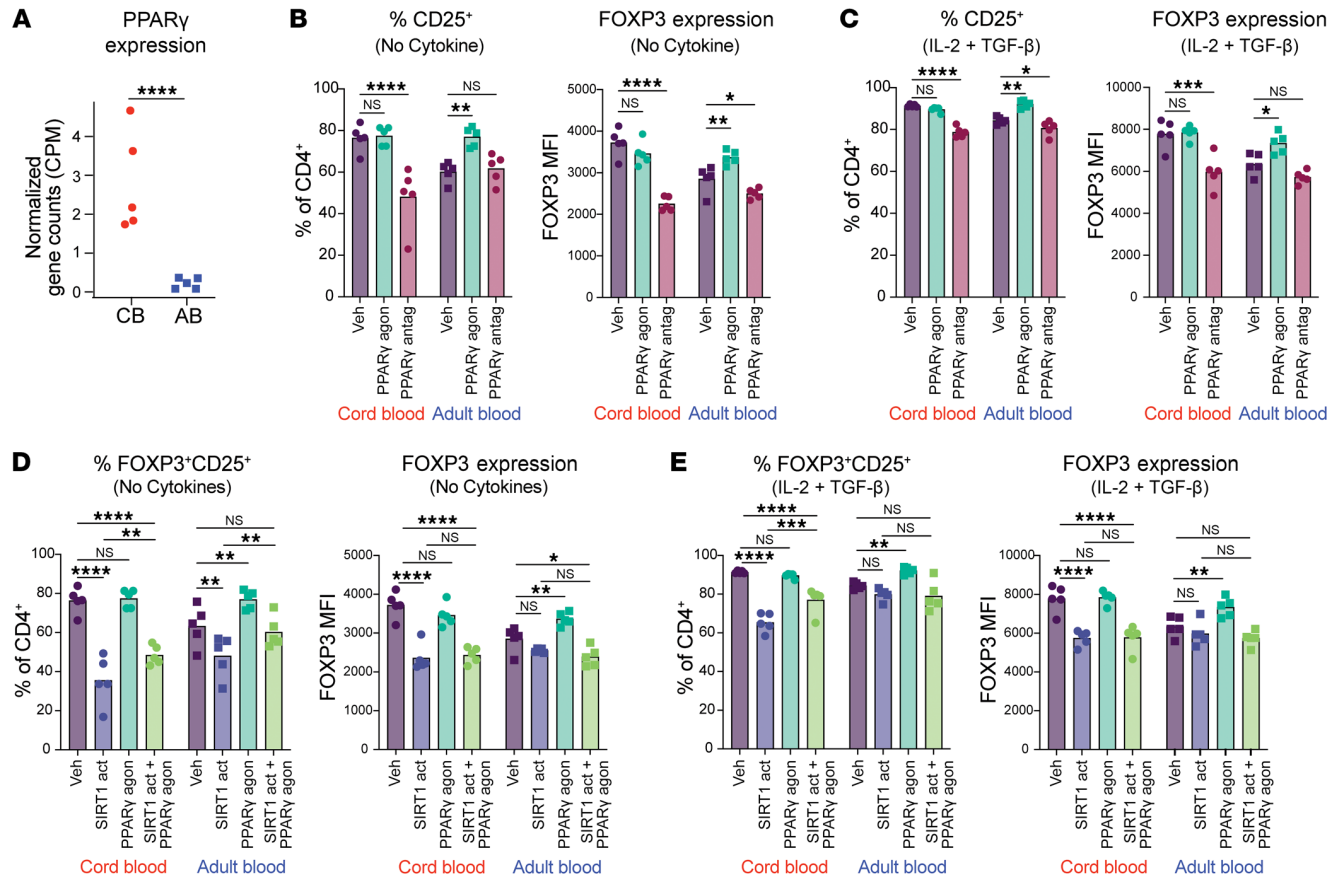
Higher PPARγ expression by neonatal naive CD4^+^ T cells facilitates their FOXP3 expression. (**A**) Normalized RNA-seq counts of PPARG 48 hours after activation. (**B**–**E**) CD25^+^ percentage and FOXP3 MFI among CB and AB naive CD4^+^ T cells treated with vehicle, PPARγ agonist or antagonist (**B** and **C**) or SIRT1 activator, PPARγ agonist, or both (**D** and **E**) before No Cytokine or IL-2+TGF-β stimulation. Data points represent individual donors. **A** was performed once. **B**–**E** show 1 of 2 experimental replicates. Unpaired *t* test was used for **A**. Two-way ANOVA with multiple comparisons used for **B**–**E**. *P ≤ 0.05, **P ≤ 0.02, ***P ≤ 0.001, ****P < 0.0001. Horizontal bars in **A** and column heights in **D**–**E** depict mean values.

In other cell types, SIRT1 negatively regulates PPARγ, with lower SIRT1 activity linked to higher PPARγ levels (47) (Figure 7A). PPARγ, a transcription factor involved in adipogenesis, lipid metabolism, and insulin sensitivity, also influences Treg biology and generation (48). RNA-seq analysis revealed PPARγ expression was 10-fold higher in stimulated CB versus AB naive CD4^+^ T cells (Figure 8A). To test its role in increased early-life Treg generation, CB and AB naive CD4^+^ T cells were treated with PPARγ agonist rosiglitazone, PPARγ antagonist GW9662, or DMSO for 24 hours before activation in No Cytokine or iTreg conditions. At 96 hours, PPARγ agonism did not affect CD25^+^ percentages or FOXP3 levels, while antagonism significantly reduced both parameters under either culture condition. In AB, PPARγ agonism increased CD25^+^ percentages and FOXP3 expression to levels approaching those in CB, whereas antagonism decreased both parameters (Figure 8, B and C). These results suggest elevated PPARγ expression in CB naive CD4^+^ T cells contributes to their enhanced capacity for FOXP3 expression.

To determine if reduced FOXP3 expression following SIRT1 activation in CB naive CD4^+^ T is primarily mediated through reduced PPARγ activity, cells were pretreated with SIRT1 activator, PPARγ agonist, or both before stimulation. SIRT1 activation alone reduced CB and AB CD25^+^ percentages and lowered CB FOXP3 levels, while adding PPARγ agonism modestly restored CB CD25^+^ percentages but failed to rescue FOXP3 expression (Figure 8, D and E). Dose titration of pharmacological agents revealed minimal effects on cell viability, activation, or proliferation, aside from a slight dose-dependent decrease in Ki-67 with SIRT1 activation. Effects of SIRT1 activation and PPARγ antagonism on CB CD25^+^ percentages were also observed at even lower concentrations (Supplemental Figure 7, D–S). These findings indicate that the CD38/NAD/SIRT1 axis influences early-life FOXP3 expression via multiple mechanisms, including direct effects on FOXP3 protein stability and metabolic state, with PPARγ as one downstream pathway. Overall, these data highlight how elevated neonatal expression of CD38 in naive CD4^+^ T cells enhances their regulatory potential and supports the early-life window for tolerance.

## Discussion

Measuring human Treg generation is challenging because markers like FOXP3, CD25, and CTLA4 are also expressed by activated effector T cells. While culture conditions with IL-2 and TGF-β have long been the gold standard for generating human iTregs, these cells display suboptimal function, reduced CNS2 demethylation, and lower FOXP3 expression stability as compared with endogenous Tregs from blood or tissue (49). Recent advances, such as targeting the RBPJ–NCOR complex to enhance FOXP3 stability (37) and optimizing ex vivo conditions to produce more stable and functional “S/F-iTregs” (36), may reinvigorate design of tolerogenic cellular therapies that do not rely on endogenous Tregs. In this context, our observation that even “traditional iTreg” conditions enable CB naive CD4^+^ T cells to develop heightened suppressive capacity, CNS2 demethylation, and FOXP3 stability when compared with their adult counterparts is particularly intriguing.

These findings raise questions about the regulation of CD38 expression in naive CD4^+^ T cells and its age-dependent shifts. PAXIP1, known to promote CD38 expression in macrophages by increasing H3K27ac at an intronic enhancer (50), has not been studied as a regulator of CD38 in T cells but does play key roles in early T cell development, including TCR gene rearrangement and thymic egress via S1PR1 expression (51). While PAXIP1 expression is not known to be age-dependent, Lin28b, an RNA-binding protein elevated in hematopoietic progenitors, influences early-life lymphocytes by enhancing TGF-β signaling critical for fetal Treg differentiation (13, 52). Lin28 also promotes glucose metabolism by inhibiting let-7 microRNA and activating MTOR (53), making it another potential regulator of age-dependent CD38 expression.

CD38 enrichment on RTEs (18) provides an intriguing explanation for why mean CD38 expression might be increased in the neonatal window, a period demarcated by high thymic output (54). A slow decrease in CD38 levels after thymic egress, combined with reduced thymic output with increasing age, could lead to this age-dependent expression pattern. We did observe higher CD38 expression in CD31^+^ compared with CD31^–^ cells in CB and AB, consistent with CD38 serving as a RTE marker. However, large age-dependent differences in CD38 levels persisted in both subsets, warranting further investigation into regulation of CD38 expression in T cells and how this relates to the age at which they were generated and their time spent in circulation. Functional studies comparing CD38^hi^ versus CD38^lo^ naive CD4^+^ T cells from AB, focusing on metabolic state and tolerogenic potential, should be prioritized in follow-up studies.

Our work underscores that the metabolic state of early-life lymphocytes fundamentally shapes functional capacity (11). Human naive neonatal CD4^+^ T cells rely more on glycolysis than adult counterparts, similar to human CD8^+^ T cells (55). In mice, neonatal CD8^+^ T cell glycolysis is linked to enhanced effector memory differentiation and reduced central memory generation (13). We show that reducing glycolysis in CB naive CD4^+^ T cells impairs their iTreg generation. Further studies are needed to clarify the mechanism linking naive CD4^+^ T cell metabolism and function, including how a high glycolytic state during stimulation of CB naive CD4^+^ T cells affects their relative expression of the *FOXP3-*E2 splice variant (56) and resulting effects of this variant on longer term iTreg stability and function (57).

An existing body of work illustrates the importance of OXPHOS in the function of terminally differentiated Tregs from adult donors (58, 59). However, glycolysis has been implicated in iTreg induction and suppressive function (56). We show that early-life naive CD4^+^ T cells are more reliant on glycolysis compared with adult cells and that this translates into differences in their tolerogenic function after activation. Thus, our study fills a gap in understanding the age-dependent differences in T cell metabolic requirements and how those differences impact emergent iTregs, rather than terminally differentiated endogenous Tregs. Additional work is needed to disentangle the longer term fate of iTregs emerging from the neonatal naive CD4^+^ T cell compartment. Development of relevant animal models, including humanized mice to track the fact of human naïve CD4^+^ T cells, will help discern if the glycolytic tone accompanying the induction of Tregs from neonatal CD4^+^ T cells impacts their long-term stability or function.

We identified SIRT1 as one downstream mediator of CD38’s influence on FOXP3 expression in neonatal naive CD4^+^ T cells. While SIRT1 activity increases later in life, mediating physiological changes associated with healthy aging (60), its role during the neonatal period remains understudied. Based on SIRT1’s wide range of acetylation targets, further investigation will be needed to fully disentangle its contributions to shaping age-dependent differences in T cell behavior, but our data implicate SIRT1-mediated FOXP3 deacetylation as one key mechanism. SIRT1 may also promote glycolysis over OXPHOS via effects on transcription factors like PGC-1α and HIF-1α or enzymes like PGAM1 (61–63). PPARγ emerges as another potential SIRT1 interactor affecting CD4^+^ T cell metabolism and Treg potential (64). SIRT1 deacetylates and inactivates PPARγ (65), so lower SIRT1 activity in neonatal naive CD4^+^ T cells may enhance PPARγ function. PPARγ also inhibits SIRT1 transcriptionally, suggesting a negative feedback loop that further restrains SIRT1 in neonatal T cells (47). CD38 was recently shown to promote stress tolerance of Tregs in the inflamed brain by limiting ADP ribosylation of CD25 despite high NAD+ levels, thereby preserving IL-2 binding (66). Whether this mechanism contributes to CD38’s role in initiation of FOXP3 expression during iTreg differentiation deserves further examination.

As for limitations of the study, assays included only 10–15 donors per age group. Full validation of all findings in CB was not possible in postnatal samples due to availability. Pharmacological agents were used because CRISPR electroporation of CB, even with a scramble control, led to poor viability and impaired activation. Despite confirming CB and AB SIRT1 expression via Western blot, attempts to measure SIRT1 activity in CB versus AB naive CD4^+^ T cells were unsuccessful. It is impossible to extrapolate the in vivo impact of the in vitro phenomena measured.

In conclusion, our findings identify CD38 expression and glycolytic state as key enablers of FOXP3 expression in neonatal naive CD4^+^ T cells, presenting potentially novel therapeutic targets to enhance early-life peripheral tolerance. This could have significant implications for preventing immune disorders such as autoimmunity and allergy. Importantly, our work underscores the need for age-specific approaches to immune modulation, as strategies to promote Treg induction in neonates may differ from those in adults. The unique neonatal immune environment, shaped by distinct metabolic cues and regulatory proteins like CD38, offers critical insights into the age-dependent plasticity of immune cells. Future studies targeting these pathways could refine interventions for neonatal immune regulation, advancing preventive and therapeutic strategies for early-life immune tolerance.

As for limitations of the study, assays included only 10–15 donors per age group. Full validation of all findings in CB was not possible in postnatal samples due to availability. Pharmacological agents were used because CRISPR electroporation of CB, even with a scramble control, led to poor viability and impaired activation. Despite confirming CB and AB SIRT1 expression via Western blot, attempts to measure SIRT1 activity in CB versus AB naive CD4^+^ T cells were unsuccessful. It is impossible to extrapolate the in vivo impact of the in vitro phenomena measured.

## Methods

### Sex as a biological variable

Both sexes were examined, and no sex-dimorphic effects were observed, though they cannot be excluded given samples size limitations.

### Sample processing

CB was shipped overnight, and AB and infant blood was processed within 12 hours of collection after rocking at room temperature (RT). PBMCs from all samples were obtained via Ficoll-Plaque gradient, then frozen at 10 × 10^7^ cells/mL in 90% FBS with 10% DMSO and stored in liquid nitrogen. Fetal spleens were digested in media supplemented with 3 mg/mL Collagenase IV and 1 mg/mL DNase I (both Sigma-Aldrich) at 37°C for 30–45 minutes in a shaking water bath. After filtering, splenic lymphocytes were isolated via Ficoll, frozen, and stored similarly.

### Cell culture and stimulation

PBMCs were thawed, washed, and rested overnight at 37°C 5% CO_2_ in Resting Media (RPMI with 10% FBS, 1% 100× Pen/Strep, 1% HEPES, and 1% Glutamax [Thermo Fischer Scientific]), then treated with the STEMCELL Technologies EasySep Human naive CD4^+^ T Cell Isolation Kit. For RNA-seq, live naive CD4^+^ T cells were sorted (CD4^+^CD8^–^CD45RA^+^CD27^+^CCR7^+^CD95^–^). Cells were analyzed immediately or activated with STEMCELL Technologies ImmunoCult Human CD3/CD28 T Cell Activator in ImmunoCult T cell Media (+0.1% Pen/Strep) at 10 × 10^5^ cells/200 μL in 96-well, U-bottom plates at 37°C 5% CO_2_ for up to 96 hours. No cytokines were added for No Cytokine conditions. iTreg conditions were adapted from prior work (4) including exogenous IL-2 (10 ng/mL; PeproTech) and TGF-β (50 ng/mL; PeproTech). In Supplemental Figure 3F, other protocols for cell activation were used: Human T-Activator CD3/CD28 Dynabeads and plate-coating with anti-CD3/CD28 antibodies (UCSF Antibody Core clone: OKT3 2 μg/mL; Miltenyi Biotec 130-093-375, clone: 15E8, 4 μg/mL) overnight at 4°C or for 4 hours at 37°C.

### ELISA

Culture supernatants were stored at –80°C and submitted to Eve Technologies for analysis using its Human Cytokine Proinflammatory-Focused 15-Plex Discovery Assay.

### SCENITH

Inhibitors, puromycin, and anti-puromycin antibodies were obtained from Rafael Argüello (Aix Marseille Université, CNRS, INSERM, CIML, Marseille, France) and used as described previously (42). Briefly, 10 × 10^5^ naive CD4^+^ T cells were incubated for 20 minutes with control (DMSO), 2DG (100 mM), oligomycin (O; 1 μM), or a combination (DGO). Puromycin (10 μg/mL) was then added for 40 (rested cells) or 15 minutes (activated cells), before cold PBS wash and cell staining.

### Seahorse

Naive CD4^+^ T cells were seeded at 150,000 cells/well on an Agilent Seahorse XFe96 analyzer using the Seahorse XF T Cell Metabolic Profiling Kit. Results were analyzed using the Agilent Seahorse Analytics web browser.

### Flow cytometry

Supplemental Table 4 contains antibody information. Surface staining was done in PBS + 2% FBS for 30 minutes at 4°C. The Foxp3 staining kit (eBioscience, 00-5523-00) was used for intracellular staining and the Annexin V Detection Kit (eBioscience, 00-8007-72) for apoptosis measurement. Events were recorded on a Fortessa (BD Biosciences) and analyzed with FlowJo.

### Pharmacological perturbations

Cells were rested for 24 hours with the following compounds at the specified final concentrations: 2DG (Sigma-Aldrich, D3179-250MG, 0.5 mM), 78c (Sigma-Aldrich, 5387630001, 5 μM), selisistat (SelleckChem, S1541, 20 μM), SRT 2104 (MedChem Express, HY-15262, 20 μM), rosiglitazone (BRL, 49653, 60 μM), GW9662 (MedChem Express, HY-16578, 2.5 μM). Cells were then either directly analyzed by Agilent Seahorse or stimulated with anti-CD3/anti-CD28 in the presence of the same chemicals.

### NAD+ measurement

CB and AB naive CD4^+^ T cells were lysed, and supernatant was assayed for total NAD^+^ using the colorimetric NAD/NADH Assay Kit (Abcam ab65348) according to manufacturer instructions. For certain studies, cells were treated with 78c or DMSO for 24 hours before lysis.

### Allogeneic MLR

CB and AB naive CD4^+^ T cells were cultured under No Cytokine or iTreg conditions for 96 hours with media change at 48 hours. CD4^+^CD127^lo^CD25^+^ or CD4^+^CD127^lo^CD25^–^ Suppressor cells were sorted from these cultures and cocultured at varying ratios with 10^5^ CTV-labeled PBMC Responder cells and 3 × 10^5^ irradiated (30 Gy) cells; the latter 2 were from separate unrelated adult donors and depleted of CD4^+^CD25^+^CD127^–^ cells. Responder cell proliferation was measured at 6–7 days.

### Stability assay

CB and AB naive CD4^+^ T cells were cultured under No Cytokine or iTreg conditions for 96 hours with media change at 48 hours. Cells were washed and rested for 96 hours with IL-2, with media change at 48 hours. Half the cells were then stained for FOXP3 and CD25 expression (first rest). Remaining cells were restimulated with ImmunoCult anti-CD3/CD28 and IL-2 for 96 hours, with media change at 48 hours. Cells were washed and rested with IL-2 for another 96 hours, with 48 hours media change, then stained (second rest). For the stability assay with 78c, 78c or DMSO was added 24 hours before the first activation and included during the first activation only.

### Th2 differentiation

Naive CD4^+^ T cells were seeded at 10 × 10^5^ cells in 200 μL media, stimulated via plate-coated anti-CD3/CD28 antibodies, and treated with IL-4 (12.5 ng/mL) and anti–IFN-γ (10 μg/mL). After 3 days, 25% of cells were moved to a new uncoated plate and treated with IL-2 (50 μg/mL), IL-4, and anti–IFN-γ. Media were refreshed on day 4. On day 5, cells were stimulated with PMA/ionomycin+Brefeldin A (BFA) and stained. For CD38 inhibition, cells were treated with 78c (5 μM) or DMSO 24 hours before Th2 differentiation.

### Demethylation

Naive CD4^+^ T cells were isolated from CB and AB (5 of each included in the first experiment, 3 of each in the second) and cultured under iTreg conditions for 96 hours with media change at 48 hours. CD4^+^CD25^+^CD127^lo^ cells were sorted from these cultures along with CD4^+^CD25^+^FOXP3^+^CD127^lo^ from AB and CB PBMCs as positive “endogenous Treg” demethylation controls. Dried cell pellets were shipped to Zymo Research Corporation for targeted bisulfite sequencing of the TSDR *FOXP3* loci. Data were reported as methylation ratios on chromosome coordinates for 15 acetylation sites, which we mapped to TSDR CpG islands (67). A linear mixed effects model was used to analyze dependence on the interactions of age (CB, AB) and CpG site (1–15), and experiment batch (batch 1, batch 2) using ‘imer’ function in nlme (v3.1.166). A random intercept was included for each patient (~1| patient). One-way ANOVA was performed with Satterthwaite’s method in the R stats package (v4.4.1). Post hoc comparisons were performed after interaction between age and CpG site was found to be significant (Pr(>F)=2.485 × 10^–6^). Pairwise test conditioned on CpG site (~ age|site) was performed using ‘emmeans’ (v1.11.1) with *P* values adjusted using Benjamini-Hochberg.

### FOXP3 acetylation

The acetylation of FOXP3 protein was assessed using the Duolink Flow Cytometry Proximity Ligation Assay (MilliporeSigma, DUO94005) according to manufacturer protocol. Naive CD4^+^ T cells from CB and AB were treated with antibodies for FOXP3 (Cell Signaling Technology, 12653) and Acetylated-Lysine (Cell Signaling Technology, 9441) at 1:100 dilution for 1 hour at RT. Duolink PLUS and MINUS PLA probes were then added, followed by ligation, amplification, and detection according to manufacturer protocol.

### Bulk RNA-seq

#### Generation of bulk RNA-seq data.

Naive CD4^+^ T cells, CD4^+^CD8^–^CD45RA^+^CD27^+^CCR7^+^CD95^–^, were sorted from CB and AB. For 0-hour point, 10 × 10^6^ cells were washed with PBS, pelleted, and frozen at –80°C. For other time points, 10 × 10^6^ cells were seeded in a 96-well plate at 10 × 10^5^ cells/well and cultured at 37°C with ImmunoCult anti-CD3/CD28 soluble antibodies; harvested at 3 hours, 24 hours, or 48 hours after activation; and processed as at the 0-hour time point. Frozen cell pellets were shipped to Novogene on dry ice, where sample quality control, library construction, and 150 cycles of paired-end sequencing were performed on an Illumina NovaSeq 6000, S4 Flowcell. Six samples for each age and time point were submitted, but only those that passed RNA quality control were advanced to sequencing. The final number of individual samples from different donors for each time point are as follows: 0HR: 6CB, 5AB; 3HR: 5CB, 5AB; 24HR: 5CB, 5AB; 48HR: 5CB, 5AB.

#### Bulk RNA-seq data processing.

cDNA and noncoding RNA FASTA files from Ensembl_GRCh38 (version 109) (68) were concatenated and input into the kallisto (0.46.2) (69) ‘index’ function with default arguments. The kallisto ‘quant’ function was used to perform paired-end transcript quantification with estimates imported into R (version 4.4.1) (70) and summarized using tximport (1.32.0) (71) (‘type=“kallisto”, countsFromAbundance=“lengthScaledTPM”, ignoreTxVersion=TRUE’) from Bioconductor (version 3.19) (72). Ensembl_IDs were mapped to gene symbols using ‘makeTxDbFromEnsembl’ from txdbmaker (1.0.1) (73) and biomaRt (74) (2.60.1) using v109 of the ‘hsapiens_gene_ensembl’ dataset. Genes with scaffold and haplotype chromosome annotations were removed before downstream analysis.

Gene counts, excluding lowly expressed genes using ‘filterByExprs,’ were used to create a ‘DEGList’ object with the group argument set to the interaction of age (CB, AB) and time point (0, 3, 24, 48 hours) using edgeR (4.2.2) (75). Normalization factors were generated using the ‘TMM’ method (76). EdgeR ‘cpm’ function with prior count of 3 was used to generate normalized log_2_ CPM as input for PCA and heatmaps after regressing on effect of sex using limma ‘removeBatchEffect’ (3.60.6) (77). Gene count plots were generated with CPM values without log transformation. PCA was performed using ‘prcomp’ in R stats (4.4.1) with ‘scale=TRUE’ on top 500 variable genes, as in the ‘plotPCA’ function from DESeq2 (1.44.0) (78). Heatmaps included 2,000 highest row variances, excluding TCR-variable and MHC-associated genes. Row transformation and ordering of heatmaps were performed using limma ‘coolmap’ with the argument ‘cluster.by=”de pattern”’. R stats ‘cuttree’ function was used to identify ‘k=8’ hierarchical gene clusters. Mean *z*-scores were used to summarize average cluster expression across time points.

#### Differential expression of bulk RNA-seq.

Differential expression and summary gene statistics were analyzed using linear models (‘lmFit’) with empirical Bayes moderation (‘eBayes’) and observational weighting (‘voomWithQualityWeights’) with limma (79). A null-intercept model accounting for group (interaction of age and time point) and sex was used for the ‘design’ argument. *P* values were adjusted using the Benjamini-Hochberg correction and ‘topTable’ function in limma. Significance thresholds were 0.585 < log_2_ fold-change and 0.05 > *P*-adj.

#### Gene set enrichment.

Gene Ontology Biological Processes pathways were obtained from the C5 collection from MSigDB (80). GSEA was performed using the fgsea package (1.30.0) with ‘scoreType=“std”’ (32), with gene sets containing at least 15 genes (minSize=15) and ranked by descending log_2_ fold-change.

#### Analysis of scRNA-seq data.

Raw counts were normalized to 1 × 10^4^ and log-transformed. Differential expression was performed using a Wilcoxon rank-sum test (sc.pp.rank_genes_groups) in scanpy (version 1.9.4) (81). Metabolic genes were curated from Human1 GEM (82).

#### Compass analysis.

Pseudobulking of scRNA-seq data was performed by averaging cell profiles from each donor. Compass was performed on central carbon pathway reactions from Human1 GEM (82). Raw reaction scores were negative-transformed and shifted as previously described (22). Variance shrinkage and linear modeling of scores were performed using limma (77).

### Mass cytometry

#### Mass cytometry antibody conjugation.

Supplemental Table 4 contains CyTOF antibodies and staining concentrations. MaxPar kits were used for conjugation (Standard BioTools 201155A). IgG concentration was measured by NanoDrop (Thermo Fisher Scientific) and metal presence confirmed by CyTOF. Antibodies were diluted to 0.2–0.5 mg/mL in stabilization buffer (Candor Bioscience, 131050) with 0.1% sodium azide.

#### Mass cytometry barcoding and staining.

Cells were thawed, washed, then rested overnight at 37°C 5% CO_2_ in Resting Media. Unless noted, all washes were performed in staining buffer (Standard BioTools, 201068) followed by a 5-minute 4°C 600*g* spin. The next day, 2.5 × 10^6^ cells/donor were resuspended in PBS+5 mM EDTA (Standard BioTools, 201058) and incubated for 60 seconds with 25 μM cisplatin (Sigma-Aldrich, P4394). Cells were washed and fixed with 1.6% paraformaldehyde (PFA; Electron Microscopy Sciences, 15710) for 10 minutes at RT. Barcoding was performed using the Cell-ID 20-Plex Palladium Barcoding Kit (Standard BioTools, 201060). Antibody cocktails were prepared in cell staining buffer with 20 mg/mL Human TruStain-FcX (BioLegend, 422302) (surface) or in Foxp3 buffer (intracellular) (eBioscience, 00-5523-00). Pooled samples were surface-stained for 30 minutes shaking at RT, washed 2 times, then fixed again with 1.6% PFA for 10 minutes at RT. Following another wash, cells were permeabilized with ice-cold methanol for 10 minutes at 4°C. Cells were washed once with staining buffer and twice with Foxp3 buffer, then intracellularly stained for 60 minutes shaking at RT. After 3 washes, cells were resuspended in PBS (Standard BioTools, 201058) containing 4% PFA and 1:1,500 191/193Ir Cell-ID Intercalator Solution (Standard BioTools, 201192A).

#### Mass cytometry data acquisition.

Cells were washed once each with cell staining media, PBS, and MilliQ water (MilliporeSigma), then filtered (Thermo Fisher Scientific, 352235) and resuspended at 1 × 10^6^ cells/mL in MilliQ water with calibration beads (Standard BioTools, 201078). Samples were run on a Helios mass cytometer (Standard BioTools).

#### Mass cytometry data analysis.

Bead-based normalization and de-barcoding were performed using Premessa (https://github.com/ParkerICI/premessa). Analyses were performed on CD45RA^+^CCR7^+^ cells manually gated in CellEngine (CellCarta Fremont) and exported as FCS files using flowCore (83). Marker expression was arcsinh-transformed using a cofactor of 5 for visualizations. Clustering was performed with CATALYST_R/Bioconductor (40) and FlowSOM (39) using these markers: CD98, CD69, LDH, PFKFB4, CS, ACADM, GLUD1, ATP5a, VDAC1, GLUT1, CytC, GAPDH, ASCT2, CPT1a, MCT1, CD38, FOXP3. The resulting 10 clusters were manually merged to 8. Cluster abundance was assessed with diffcyt (84) using a generalized linear model with age as a fixed effect and donor as a random effect.

### Statistics

Unless otherwise noted, statistics were calculated in GraphPad Prism. Unpaired 2-tailed *t* tests were used for 2-way comparisons of unrelated samples and 2-tailed paired *t* tests for comparison of identical samples under 2 conditions. Bonferroni’s adjustment was used where appropriate. One-way ANOVA with multiple comparisons was used for ≥3 comparisons of unrelated biological samples, and a 1-way repeated measures ANOVA with multiple comparisons was used for identical samples under various treatments or at different time points. Specific tests are noted in figure legends. An adjusted *P* < 0.05 was considered significant.

### Study approval

Deidentified human CB samples from healthy term pregnancies were obtained from the Umbilical Cord Blood Collection Program (IRB796298), made available under the California Health & Safety Code §§ 1627–1630, and maintained by the Institute for Regenerative Cures, University of California, Davis. AB from healthy, nonobese, nonsmoking donors aged 18–45 was obtained under University of California, San Francisco, IRBs 12-09489 and 13-11307. Fetal spleens were obtained from pregnancy terminations with maternal written consent, in accordance with institutional and federal guidelines, excluding cases of known maternal infection, intrauterine demise, or suspected chromosomal abnormality. Preterm infant blood samples were obtained from the Prediction of Maturity, Morbidity, in Preterm Infants (PROMPT) study, approved by the Western Institutional Review Board 20202748, an independent review board based in Puyallup, Washington, with consent from maternal individuals. Pediatric blood samples were obtained through study 05-041K, 06-149 approved by the University Medical Center Utrecht. All individuals were consented in writing for participation.

### Data availability

Graphs were made in GraphPad Prism 10, based on data included in the Supporting Data Values file. Bulk RNA-seq data from this study are available on NCBI GEO GSE282496.

## Author contributions

LRD and TCS designed the studies and wrote the manuscript. LRD performed experiments and analyzed the data. AMD performed CyTOF experiments and analysis. SC performed bulk RNA-seq analysis. VG assisted in experimental design and execution. CC performed Compass analyses. EER, SPO, and LLJP provided access to preterm samples. TVDB and FVW provided access to healthy pediatric samples. SVL, RLR, AW, and AJC provided resources and input on experimental design. TCS oversaw all study design and data analysis. All authors discussed results and commented on the manuscript.

## Conflict of interest

TCS is on the Scientific Advisory Board of Concerto Biosciences. LLJP and SPO have a patent pending (2021-058-1) for a newborn metabolic vulnerability model for identifying preterm infants at risk of adverse outcomes. SVL is a board member, consultant, and stockholder of Siolta Therapeutics and consults for Sanofi and the Atria Institute. AJC receives research support from Eli Lilly and Genentech (Roche).

## Funding support

This work is the result of NIH funding, in whole or in part, and is subject to the NIH Public Access Policy. Through acceptance of this federal funding, the NIH has been given a right to make the work publicly available in PubMed Central.

- NIH grants: R01HD102381, P30DK063720, 1S10ODO18040, P30DK098722, T32AI007334-33, 1F31AI186359, and 5T32AR007175-48.
- UCSF BCMM Trainee Research Award.
- Startup funds from UCSF’s Department of Dermatology.
- UCSF ImmunoX CoProject award.

## Supplementary Material

Supplemental data

Supplemental table 1

Supplemental table 2

Supplemental table 3

Supplemental table 4

Supporting data values

## Version 1 02/26/2026

In-Press Preview

## Version 2 04/15/2026

Electronic publication
