# Supplementary material for: CD38 expression by neonatal human naive CD4+ T cells shapes their distinct metabolic and tolerogenic properties
Source: J Clin Invest. 2026 Feb 26;136(8):e200062. doi: 10.1172/JCI200062 (PMC13078883; doi:10.1172/JCI200062)
Supplement: Supplemental data [file jci-136-200062-s245.pdf]

Supplementary Figures

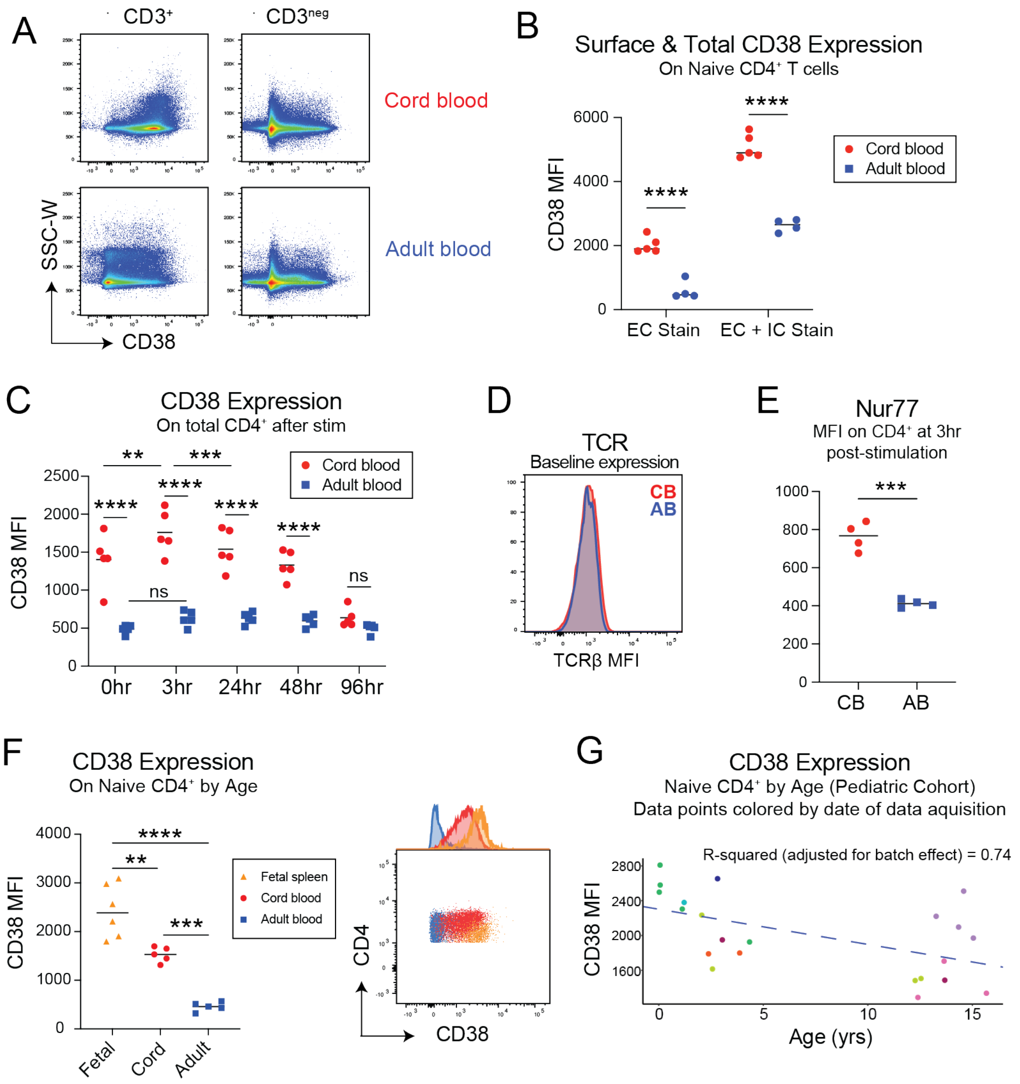

Figure S1: Related to main Figure 1 (A) Staining of CD38 on CD3$^+$ and CD3$^-$ cells from cord blood (CB) and adult blood (AB) at baseline. (B) CD38 MFI on CB and AB naïve CD4$^+$ T cells when stained extracellularly (EC) vs extra- and intracellularly (EC + IC). (C) CD38 MFI on CD4$^+$ T cells at 0, 3, 24, 48, and 96hr after activation with Immunocult^{TM} anti-CD3/CD28 soluble antibodies. (D) TCRβ MFI on naïve CD4$^+$ T cells from CB and AB measured at baseline (0hr). (E) Nur77 mean fluorescence intensity (MFI) in CD4$^+$ T cells measured 3hr post stimulation. (F) CD38 MFI on naïve CD4$^+$ T cells from 21-24 gestational week fetal spleen, CB, or AB. (G) CD38 MFI on naïve CD4$^+$ T cells from peripheral blood of healthy pediatric patients undergoing elective surgery. Data points colored by date/batch of flow cytometry data acquisition. Linear regression analysis included an adjustment for batch effect. Two-way ANOVA was used for statistics in B, C, F and unpaired t-test for E, p-values are represented as follows: * ≤ 0.05, ** ≤ 0.02, *** ≤ 0.001, **** < 0.0001.

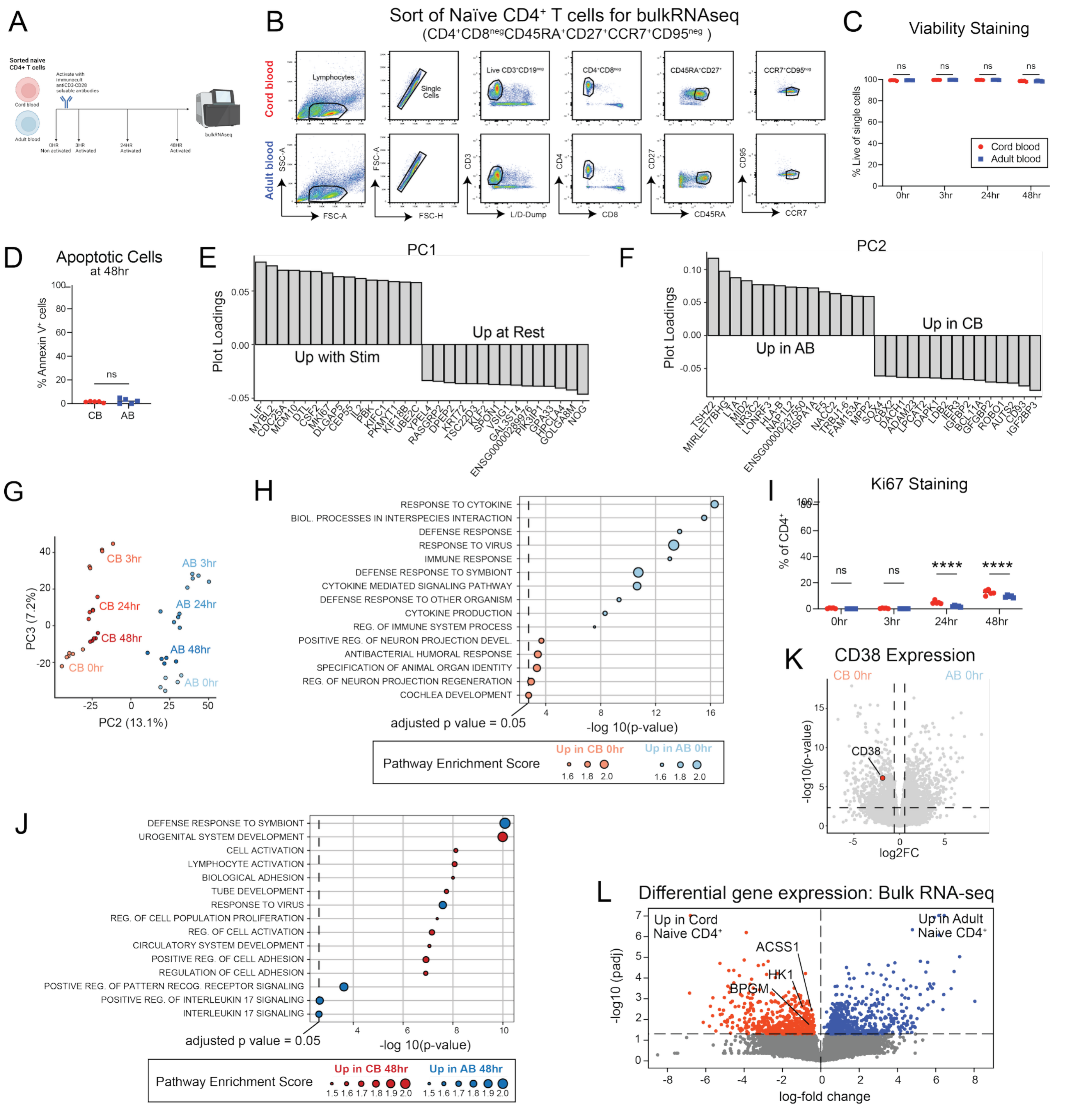

Figure S2: Related to main Figure 2 (A) Schematic of bulk RNA-seq data acquisition. CB and AB naïve CD4$^+$ T cells were isolated via FACS and sequenced at 0hr (baseline, non-activated) or following 3hr, 24hr or 48hr of activation with ImmunoCult^{TM} anti-CD3/CD28 soluble antibodies. (B) Sort strategy for naïve CD4$^+$ T cells from CB and AB for bulk RNA-seq (Live CD4$^+$CD8^{neg}CD45RA$^+$CD27$^+$CCR7$^+$CD95^{neg}). (C-D) Separately, naïve CD4$^+$ T cells from CB and AB were isolated via EasySep™ Human Naïve CD4$^+$ T Cell Isolation Kit and subjected to analogous TCR stimulation. (C) Percent of live cells after 0hr, 3hr, 24hr and 48hr of activation based on viability dye staining. (D) Annexin V staining (eBioscience™ Annexin V Apoptosis Detection Kit) at 48hr. (E) Gene loadings with the greatest contributions to PC1 are associated with activation. (F) Gene loadings with the greatest contributions to PC2 are associated with age. (G) PC2 vs. PC3 from bulk RNA-seq dataset. (H) Gene set enrichment analysis on ranked genes in CB at 0hr and 48hr. (I) CB and AB naïve CD4$^+$ T cells were isolated and stimulated as in C-D. Flow staining for Ki67 at 0hr, 3hr, 24hr and 48hr. (J) Gene set enrichment analysis on ranked genes in AB at 0hr and 48hr. (K) Volcano plot highlighting CD38 from RNA-seq of CB versus AB naive CD4$^+$ T cells at 0hr. (L) Differential expression analysis on bulk RNA-seq data shows upregulation of certain glycolysis-related genes. Genes with statistically significant differential expression are colored by age.

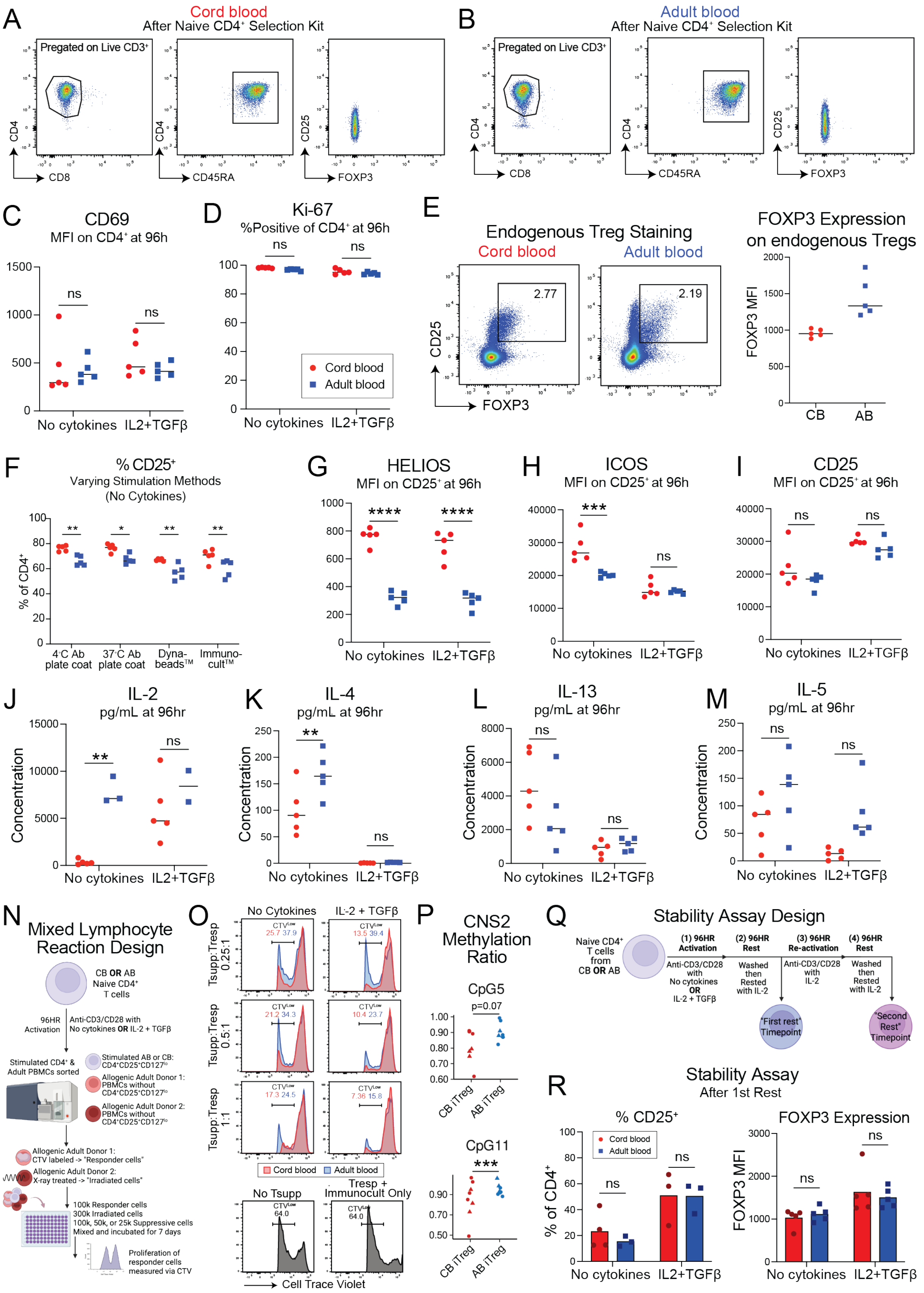

Figure S3: Related to main Figure 3 (A,B) Representative flow plots showing the EasySep™ Human Naïve CD4+ T Cell Isolation Kit results in a naïve CD4+ T cell population after use from cord blood (CB) and adult blood (AB) peripheral blood mononuclear cells (PBMCs). (C, D) CD69 and Ki67 on total CD4+ population, after naïve CD4+ T cell were isolated from CB and AB and profiled at 96hr after TCR stimulation with Immunocult™ anti-CD3/CD28 soluble antibodies with no added cytokines or with IL-2 (10 ng/ml; PeproTech) and TGF-β (50 ng/ml; PeproTech). (E) Staining patterns for FOXP and CD25 expression on endogenous Tregs from CB and AB whole PBMCs and quantification of FOXP3 MFI on populations gated at left. (F) Percentage of CD25+ cells from CB and AB naïve CD4+ T cells at 96hr after

TCR stimulation by different methods, including culture in a plate coated with anti-CD3/CD28 antibodies overnight at 4°C, in a plate coated with anti-CD3/CD28 antibodies for 4hr at 37°C, with beads added to culture coated in anti-CD3/CD28 (Dynabeads™), or with Immunocult™ anti-CD3/CD28 soluble antibodies. All with no added cytokines. (G-I) Expression of various markers on $CD25^{+}$ at 96hr after CB or AB naïve $CD4^{+}$ T cell stimulation, as measured by mean fluorescence intensity (MFI) by flow cytometry. (J-M) ELISA-based measurement of cytokines in cell culture supernatant of stimulated CB and AB naïve $CD4^{+}$ T cells at 96hr. (N) Schematic of mixed lymphocyte reaction ("Allo-MLR") where CB and AB naïve $CD4^{+}$ T cells were activated with Immunocult^{TM} anti-CD3/CD28 soluble antibodies and cultured for 96hr under no cytokine or IL-2+TGF-β conditions. Cells were then sorted to obtain $CD4^{+}CD25^{+}CD127^{LO}$ cells, "Suppressor cells". Two unrelated AB donors' PBMCs were thawed and sorted to remove $CD4^{+}CD25^{+}CD127^{LO}$ cells from those samples. One was stained with CTV ("Responder cells") while the other was irradiated ("Irradiated cells") to serve as a source of antigen-presenting cells. Cells were then mixed together at the ratio of 100,000 Responder cells, 300,000 Irradiated cells, and 100,000, 50,000, or 25,000 Suppressive cells with no added anti-CD3/CD28 stimulation. Proliferation was measured via CTV after 7 days of culture. (O) Cell Trace Violet (CTV) expression from Allo-MLR cultures showing proliferation levels of Responder cells between conditions. Responder cells with Irradiated cells (without Suppressor Cells) as well as Responder cells with ImmunoCult and no Suppressive or Irradiated cells were used as controls. (P) Comparisons in methylation ratio between CB and AB for specific CpG sites. (Q) Schematic of stability assay. FOXP3 and CD25 expression stability was assessed in CB and AB where naïve $CD4^{+}$ T cells were activated with anti-CD3/CD28 soluble antibodies and cultured for 96hr under no cytokine or IL-2+TGF-β conditions. Cells were then washed to remove stimulation and cytokines and rested with 96hr with IL-2 in all conditions. After the first 96hr rest, half the cells were removed to assess FOXP3 and CD25 expression. Remaining cells were then re-stimulated with anti-CD3/CD28 soluble antibodies and IL-2 for 96hr. Following the second stimulation, cells were washed and rested with IL-2 for 96hr. After this second rest, FOXP3 and CD25 expression were re-assessed. (R) Percentage of $CD25^{+}$ cells and their FOXP3 expression after the first stability assay rest. Two-way ANOVA was used for statistics except for P, where a linear mixed model was used, p-values are represented as follows: * ≤ 0.05, ** ≤ 0.02, *** ≤ 0.001, **** < 0.0001.

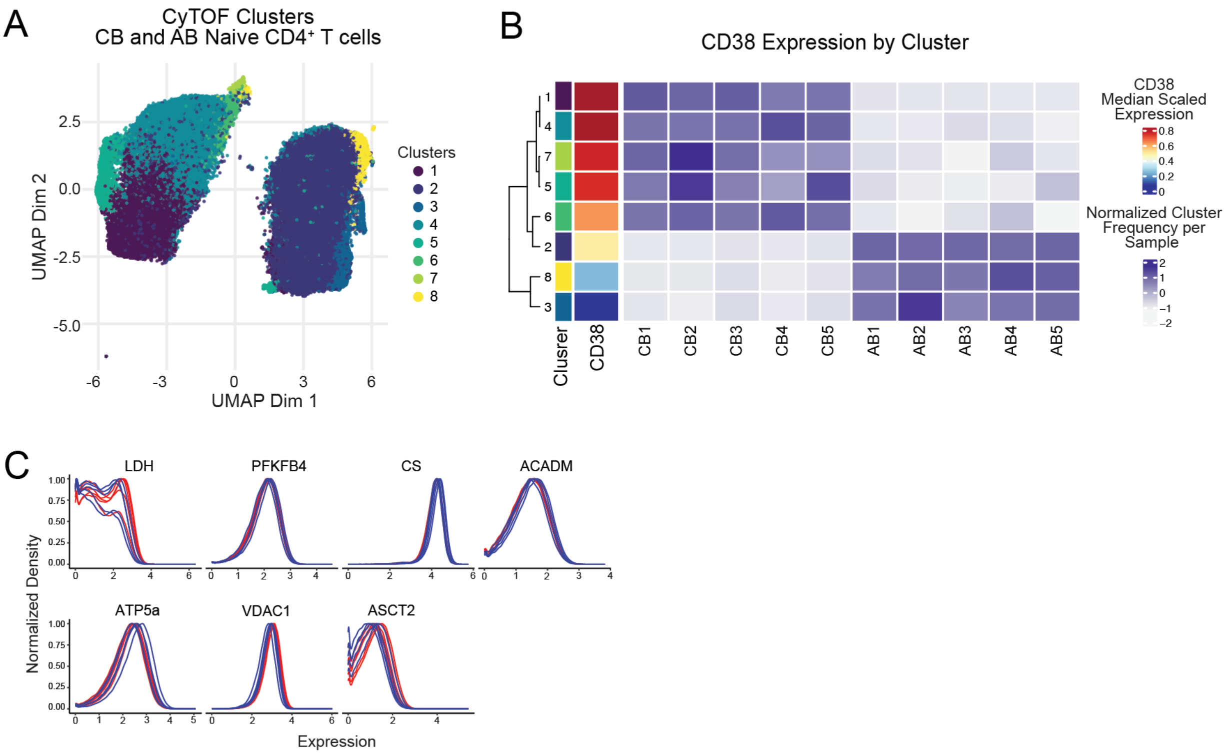

Figure S4: Related to main Figure 4 (A) To examine the different metabolic states present in the naïve CD4+ T cell pool across ages, clustering was performed with the FlowSOM algorithm using metabolic and reduced lineage markers, yielding 8 distinct clusters. (B) CD38 expression across the 8 distinct clusters in CB and AB naïve CD4+ T cells. (C) Expression patterns of metabolic markers for all CB and AB donors.

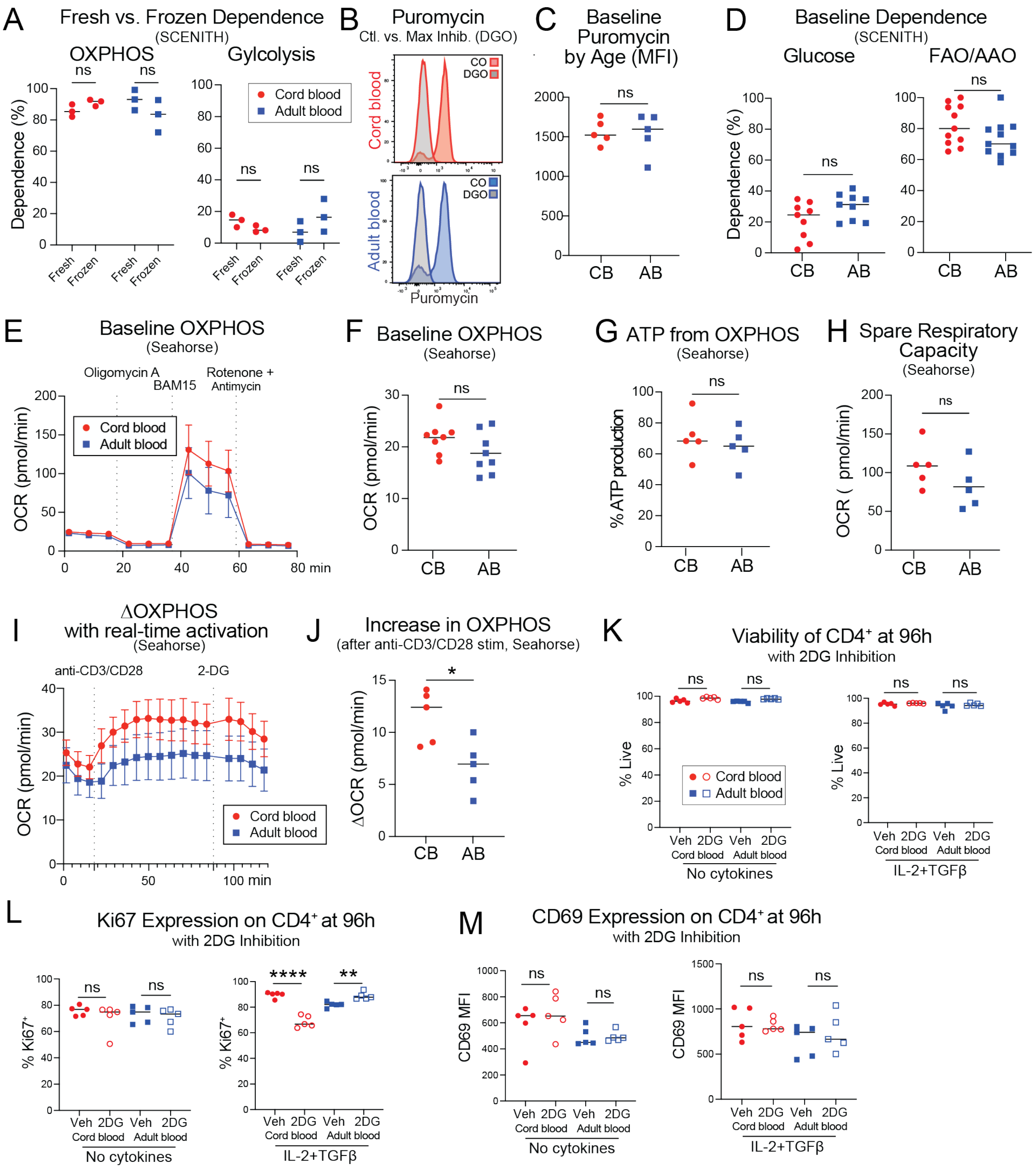

Figure S5: Related to main Figure 5 (A) Baseline (non-activated) dependence on glycolysis and mitochondrial oxidative phosphorylation in fresh and previously frozen cord and adult CD4⁺ T cells measured via SCENITH. (B) Example staining for SCENITH of puromycin MFI shift with Control vs DGO conditions for baseline (non-activated) CB and AB naive CD4⁺ T cells. (C) Quantification of puromycin MFI in Control (no metabolic inhibitor condition) for baseline (non-activated) CB and AB naive CD4⁺ T cells. (D) Baseline (non-activated) dependence on glucose and fatty acid/amino acid oxidation in CB and AB naive CD4⁺ T cells measured via SCENITH. (E) Seahorse graph of OCR (oxidative phosphorylation, OXPHOS) of baseline (non-activated) CB and AB naive CD4⁺ T cells. (F) Quantification of baseline OXPHOS between CB and AB naive CD4⁺ T cells via Seahorse. (G) The percent of ATP made from OXPHOS between CB and AB naive CD4⁺ T cells via Seahorse. (H) Spare respirator capacity between CB and AB naive CD4⁺ T cells via Seahorse. (I) Seahorse graph of ECAR after acute injection with anti-CD3/CD28 soluble antibodies between CB and AB naive CD4⁺ T cells via Seahorse. (J) The change in OCR after acute injection with anti-CD3/CD28 soluble antibodies between CB and AB naive CD4⁺ T cells via Seahorse. (K-M) Quantification of total live cells (K), Ki67⁺ (L), and CD69 MFI (M) on CD4⁺ T cells after 24 hours of treatment with 0.5mM 2DG followed by activation with anti-CD3/CD28 soluble antibodies with no added cytokines or with IL-2 + TGF-β, measured 96 hours after activation. Two-way ANOVA was used for statistics in A, K-M and unpaired t-test used for C, D, F-H, and J, p-values are represented as follows: * ≤ 0.05, ** ≤ 0.02, *** ≤ 0.001, **** < 0.0001.

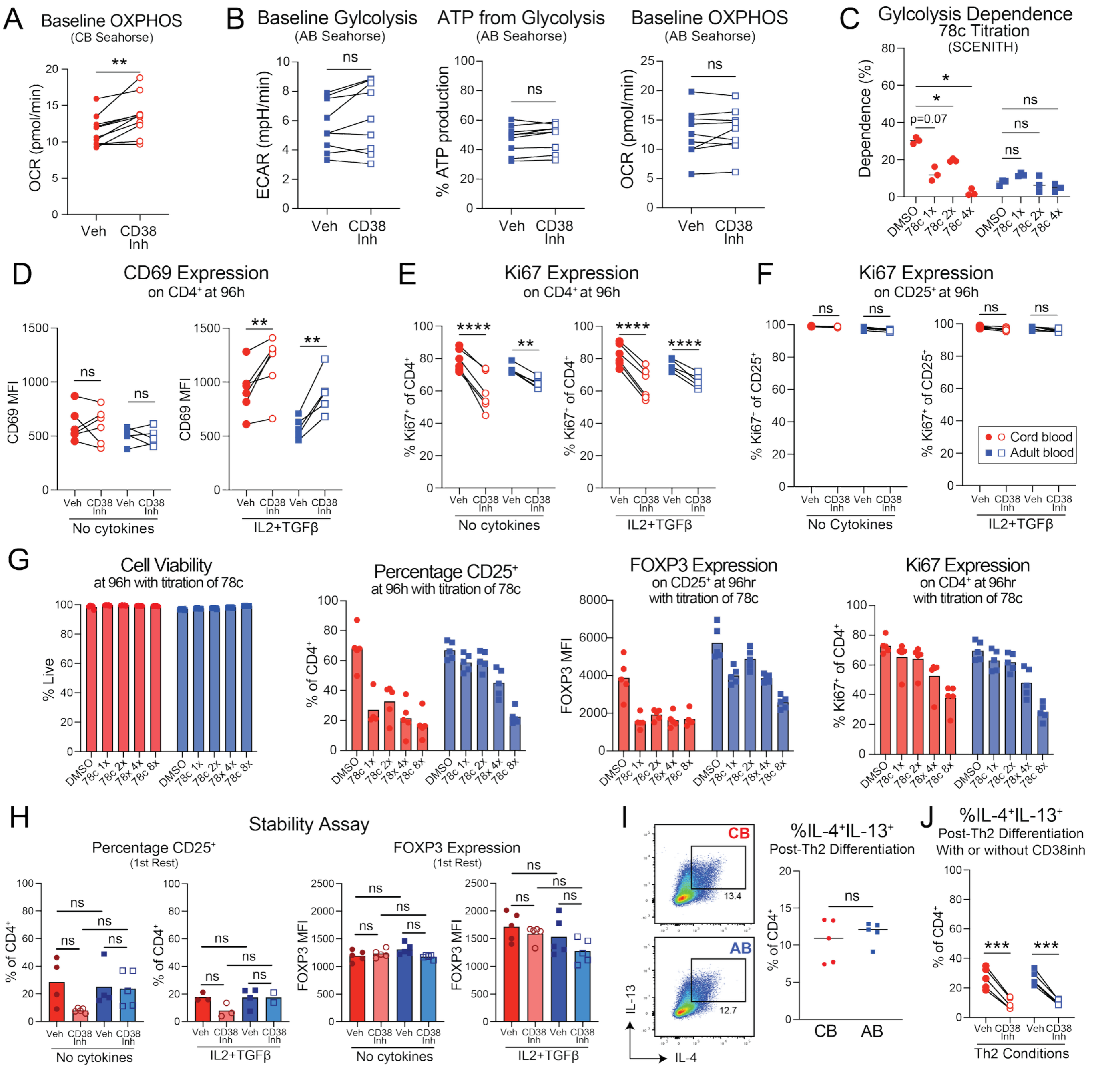

Figure S6: Related to main Figure 6 (A) (Baseline (non-activated) OXPHOS in CB naïve CD4$^+$ T cells after 24hr of 5uM 78c treatment measured via Seahorse. (B) Baseline (non-activated) glycolysis, ATP from glycolysis, and OXPHOS in AB naïve CD4$^+$ T cells after 24hr of 78c treatment measured via Seahorse. (C) Glycolytic dependence measured via SCENITH in CB and AB naïve CD4$^+$ T cells after 24 hours of treatment with various doses of 78c (1x= 5uM). (D-F) Quantification at 96 hour post-stimulation of CD69 MFI on total CD4$^+$ (D), Ki67$^+$ on total CD4$^+$ (E), and Ki67$^+$ on CD25$^+$ (F) after 24 hours of pre-treatment with 5uM 78c followed by “No Cytokine” or “IL-2 + TGF-β” activation in the ongoing presence of 78c. (G) Cell viability, percent CD25$^+$, FOXP3 MFI on CD25$^+$, and percent Ki67$^+$ in the setting of “No Cytokine” stimulation with various doses of 78c (1x = 5uM). (H) Percentage CD25$^+$ cells and their FOXP3 expression following the first rest of Treg stability assay with 5uM 78c added for 24hr before activation and during the first activation. (I) IL-4 and IL-13 expression was measured from Th2 differentiation of naïve CD4$^+$ T cells from CB and AB. (J) IL-4 and IL-13 expression was measured from Th2 differentiation of naïve CD4$^+$ T cells from CB and AB where 5uM 78c was added for 24hr prior to and during the Th2 differentiation. Unpaired t-test was used for statistics in A, I, paired t-test was used for B, F, J, and two-way ANOVA with multiple comparisons for D, E, p-values are represented as follows: * ≤ 0.05, ** ≤ 0.02, *** ≤ 0.001, **** < 0.0001.

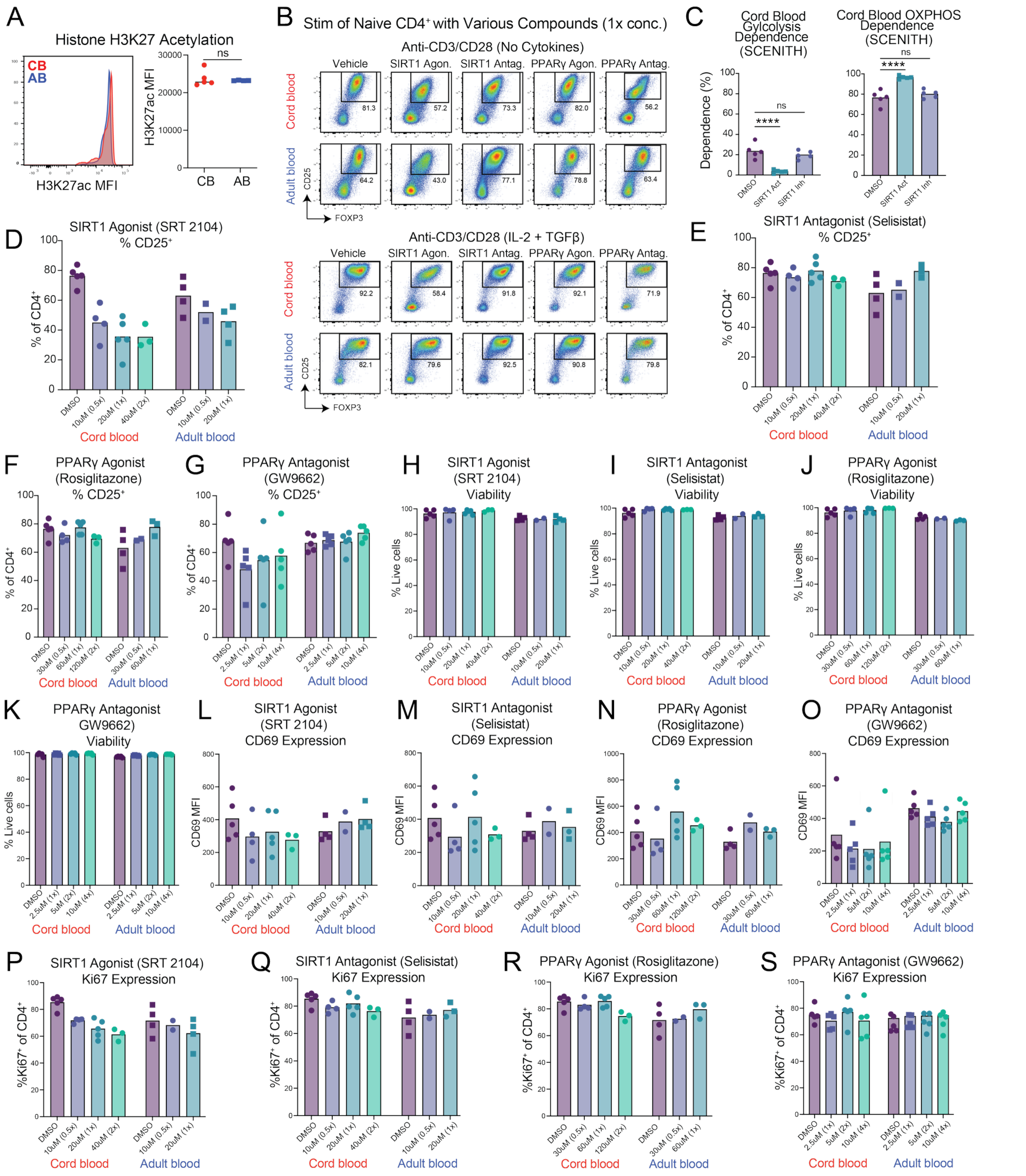

Figure S7: Related to main Figures 7 & 8 (A) H3K27ac staining of CB and AB naïve $CD4^+$ T cells using an antibody against Acetyl-Histone H3 (Lys27) (Cell Signaling Tech, # 39030S). (B) Flow plots of $CD25^+$ cells from CB and AB "No Cytokine" and "IL-2 + TGF-β" culture conditions treated with DMSO (Vehicle) or pharmacological compounds as specified in the figure. (C) CB naïve $CD4^+$ T cells were isolated and treated with vehicle control (DMSO), SIRT1 activator (20uM of SRT 2104), or SIRT1 inhibitor (20uM of Selisistat) for 24hr before performing SCENITH to assess metabolic impacts. (D-S) Quantification of $CD25^+$ percentages (D-G), cell viability (H-K), activation as marked by CD69 MFI (L-O) or recent proliferation as indicated by percentage of $Ki67^+$ (P-S) from CB and AB "No Cytokine" culture conditions treated with DMSO (vehicle control) or various doses of several pharmacological compounds. Unpaired t-test was used for statistics in A and E, paired t-test for B, and two-way ANOVA with multiple comparisons was used for C, p-values are represented as follows: * ≤ 0.05, ** ≤ 0.02, *** ≤ 0.001, **** < 0.0001.
